# Photochemical dearomative skeletal modifications of heteroaromatics

**DOI:** 10.1039/d4cs00137k

**Published:** 2024-05-31

**Authors:** Peng Ji, Kuaikuai Duan, Menglong Li, Zhiyuan Wang, Xiang Meng, Yueteng Zhang, Wei Wang

**Affiliations:** a Department of Pharmacology and Toxicology, R. Ken Coit College of Pharmacy, University of Arizona USA weiwang1@arizona.edu; b Department of Chemistry and Biochemistry, University of California, San Diego 9500 Gilman Drive La Jolla California 92093 USA peji@ucsd.edu; c Tri-institutional Center for Translational Research in Neuroimaging and Data Science (TReNDS), Georgia State University, Georgia Institute of Technology, Emory University Atlanta USA; d Tianjian Laboratory of Advanced Biomedical Sciences, Academy of Medical Science, School of Basic Medicinal Sciences, Zhengzhou University Zhengzhou Henan 450001 China yuetengzhang@zzu.edu.cn; e Henan Institute of Advanced Technology, Zhengzhou University Zhengzhou 450001 China

## Abstract

Dearomatization has emerged as a powerful tool for rapid construction of 3D molecular architectures from simple, abundant, and planar (hetero)arenes. The field has evolved beyond simple dearomatization driven by new synthetic technology development. With the renaissance of photocatalysis and expansion of the activation mode, the last few years have witnessed impressive developments in innovative photochemical dearomatization methodologies, enabling skeletal modifications of dearomatized structures. They offer truly efficient and useful tools for facile construction of highly complex structures, which are viable for natural product synthesis and drug discovery. In this review, we aim to provide a mechanistically insightful overview on these innovations based on the degree of skeletal alteration, categorized into dearomative functionalization and skeletal editing, and to highlight their synthetic utilities.

## Abstract 2

Dearomatization has emerged as a powerful tool for rapid construction of 3D molecular architectures from simple, abundant, and planar (hetero)arenes.

## Graphical abstract

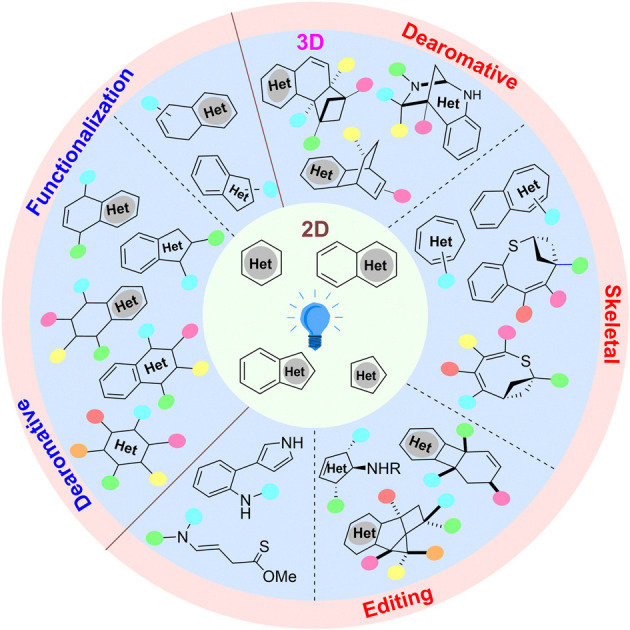

## 1 Introduction

Aromatic compounds including heteroaromatics are one of the most abundant chemical feedstocks and are broadly used in organic synthesis.^1^ Dearomatization of planar (hetero)aromatic structures offers a cost-effective and straightforward approach to non-planar molecular architectures, which are highly valued in synthesis and drug discovery.^2–4^ Various synthetic technologies derived from Birch reduction and transition-metal catalysed hydrogenation have been developed for direct reductions of aromatic nuclei including asymmetric hydrogenation^5^ and electrochemical^6,7^/photochemical Birch-type reactions^8^ under mild conditions.^9^ The drawback of these dearomative reduction methods is that it is generally difficult to install functionalities. Pre-functionalized heteroarenes are often used.^10^

**Scheme 1:**
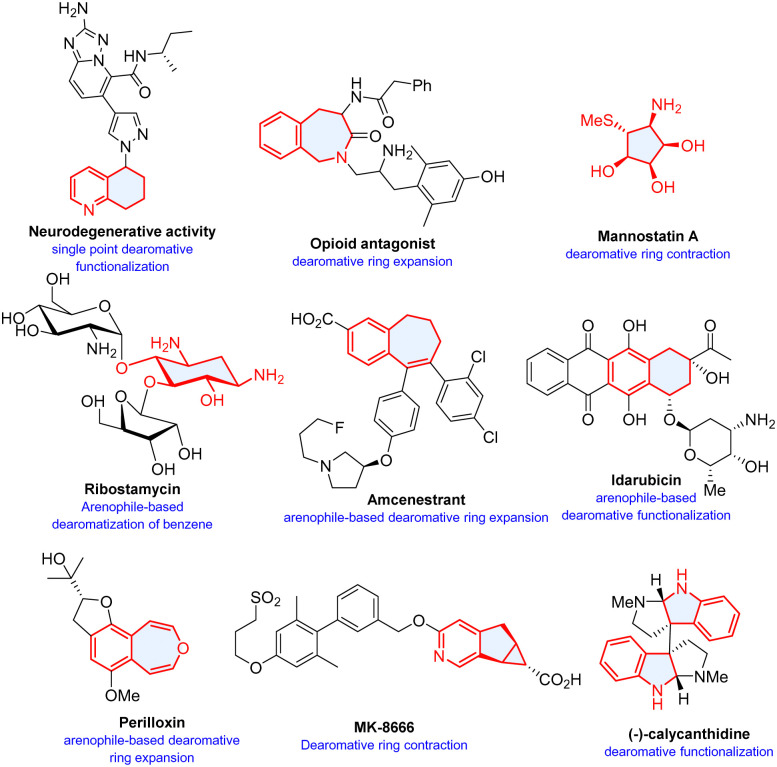
Selected examples of bioactive molecules or natural products synthesized by dearomatization. The cycles filled with light blue colour represent structures synthesized through dearomative skeletal modifications.

Photochemical reactions have long been used for dearomatization of arenes. Although arenes are highly sensitive to ultraviolet (UV)-light,^11,12^ the relatively harsh reaction conditions often result in poor selectivity and narrow functional group tolerance. Mild visible light mediated^13–16^ photochemical dearomatization has surged significantly in the recent past and offers versatile approaches to natural products and pharmaceutically valued molecules (Scheme 1).^17–19^ Multitudinous excellent reviews have highlighted the advances from different aspects. Sarlah's account focuses on unactive arenes,^20^ You and coworkers summarize diverse visible-light-induced dearomatization of indoles and nonactivated arenes and their synthetic applications.^21^ In particular, You conceptualized catalytic asymmetric dearomatization (CADA) by implementing transition-metal and organo-catalysis, and recently photo-catalysis.^22–28^ A recent review by Sánchez-Roselló and Carlos del Pozo summarizes nucleophilic dearomatization of pyridines, quinolines, and isoquinolines.^29^

**Scheme 2:**
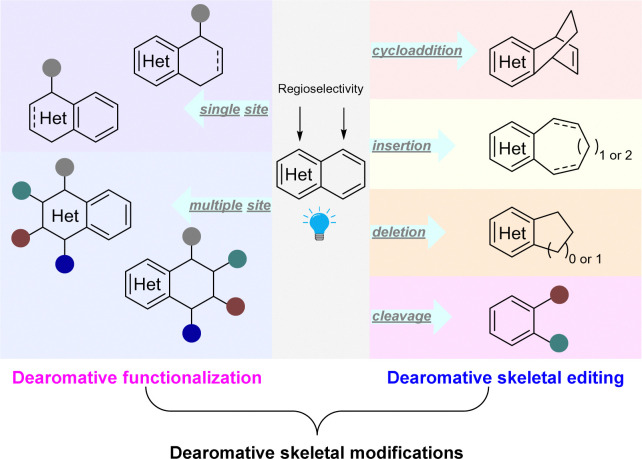
State-of-the-art synthetic approaches for dearomative skeletal modifications of heteroarenes.

Recently there has been significant development in visible-light mediated dearomatization of heteroarenes. Notably, in addition to indoles, pyridines, (iso)quinolines, benzothiophenes, benzofurans, thiophenes, furans, and pyrroles have been validated as viable substrates for these processes. Furthermore, in contrast to Birch reduction and transition-metal catalysed hydrogenation, the photocatalytic dearomatization methods enable functionalization and skeletal editing of the heteroarene scaffolds. Thus, highly complex, functional and/or scaffold diversified molecular architectures are efficiently constructed. These dearomative heterocyclic structures are particularly attractive in medicinal chemistry and drug discovery.^2–4^ Despite the fact that the transition-metal catalysed dearomatization of heteroarenes and funtionalizations have been extensively studied,^10,29,30^ photochemical dearomative skeletal remodelling only occurred recently. Herein, we summarize the fast-paced developments in the past few years. Based on the format of dearomative skeletal modifications of parent heterocycles, we categorized these approaches into (1) dearomative functionalization and (2) dearomative skeletal editing (Scheme 2). The former case retains the ring size of the core skeleton unchanged after dearomative peripheral editing of heteroarenes by installation of one or multiple functional groups, whereas the latter strategy alters the original heterocycle frameworks after dearomatization through photocycloaddition, ring expansion, ring contraction, and ring cleavage.

## 2 Photochemical dearomative functionalization of heteroaromatics

**Scheme 3:**
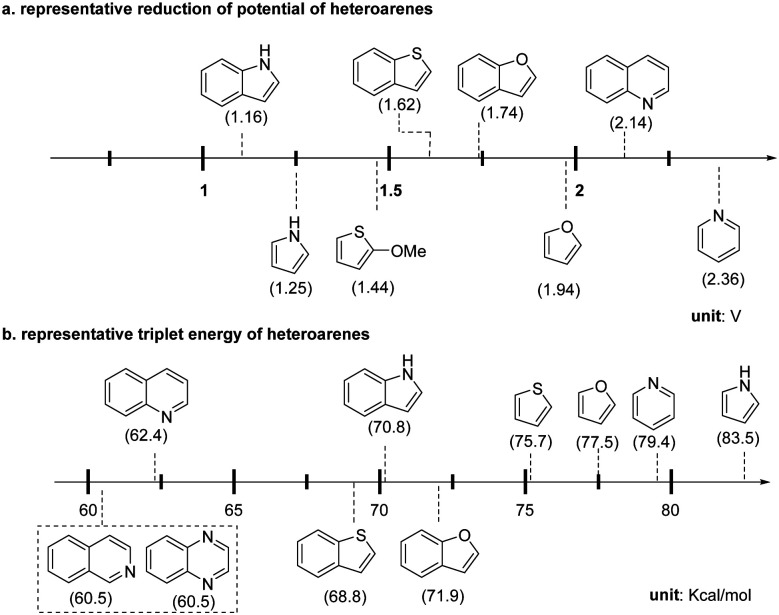
Physiochemical properties of representative heteroarenes.

The facile construction of highly functionalized and saturated/semi-saturated poly-heterocycles is critical in synthesis and drug discovery.^1,17,31^ Light-mediated dearomative functionalization offered an efficient way to build up such synthetically challenging molecular architectures. Herein, we will discuss the photochemical dearomative functionalization reactions in this section. Based on the degree of dearomative modifications on the heteroaromatics, we divide these methods into two categories: single and hydro-functionalization, and multiple-point dearomative functionalization. To compare the physiochemical characteristics of different heteroaromatics for rationalization of the chemical process, the representative redox potential and triplet energy of common heteroarenes are illustrated in Scheme 3.

### 2.1 Single-point dearomative functionalization of heteroaromatics

In this section, we focus on the methodologies, which install single functionality on the dearomatized heteroarenes commonly involving one C–H bond and one new C–X (X ≠ H) chemical bond formation, *e.g.*, C(sp^3^)–N, C(sp^3^)–C, C(sp^3^)–Si, and C(sp^3^)–B. Mechanistically, the single site dearomative functionalization of heteroarenes usually requires one hydrogen atom transfer (HAT), proton transfer (PT), and protonation process to promote new C–H bond formation. Another recurrent mechanism for single point dearoatization of indole involves new C–C bond generation and double bond isomerization after deprotonation. Based on different electronic properties of heteroarenes, a series of photoredox catalysed dearomative nucleophilic addition, nucleophilic/amphiphilic radical addition, and dearomative radical-spirocyclization processes, have been elegantly realized for single-point dearomative functionalization.

#### 2.1.1 Umpolung strategy enabled selective dearomative functionalization of bicyclic azoarenes

**Scheme 4:**
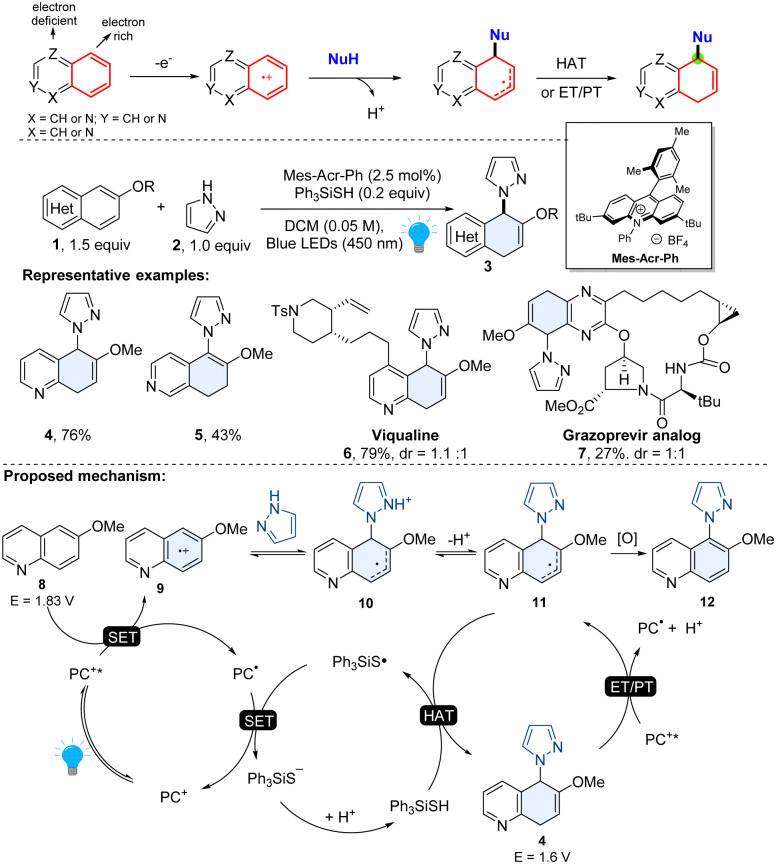
Umpolung strategy enabled dearomative functionalization of bicyclic heteroarenes.

Among heteroaromatics, selective dearomative functionalization of the phenyl moiety in bicyclic azoarenes (quinoline, isoquinoline, quinazoline, quinoxaline) is quite challenging, presumably due to a lack of a way to selectively activate the inert phenyl group. In 2022, Wang and coworkers introduced a general organophotoredox approach for the chemo- and regio-selective dearomatization of the electron rich phenyl group of diverse bicyclic heteroaromatics.^32^ The regioselectivity of this method relied on the precise manipulation of the electronic nature of bicyclic heteroarenes. As shown in Scheme 4, compared to electron deficient heterocyclics, the phenyl moiety of bicyclic azoarenes is relatively electron-rich. It is believed that it tends to undergo discriminatory oxidative single electron transfer (SET) using an excited photocatalyst. The resulting reactive radical cation intermediate 9 could be attacked by nucleophilic azoles or primary amines. After the subsequent deprotonation, the radical intermediate 11 grasps a hydrogen atom from the hydrogen atom transfer (HAT) agent thiol and produces 5,8-dihydroquinoline (*e.g.* compound 4) or 7,8-dihydroisoquinoline (*e.g.* compound 5). This method tolerates various functional groups and is applicable to many common polycyclic arenes including (iso)quinoline, quinoxaline, naphthalenes, anthracenes, and phenanthrenes. The utility of this method was showcased by the late-stage functionalization of complex pharmaceutically related molecules (6–7) and the rapid synthesis of bioactive molecules.^32^

**Scheme 5:**
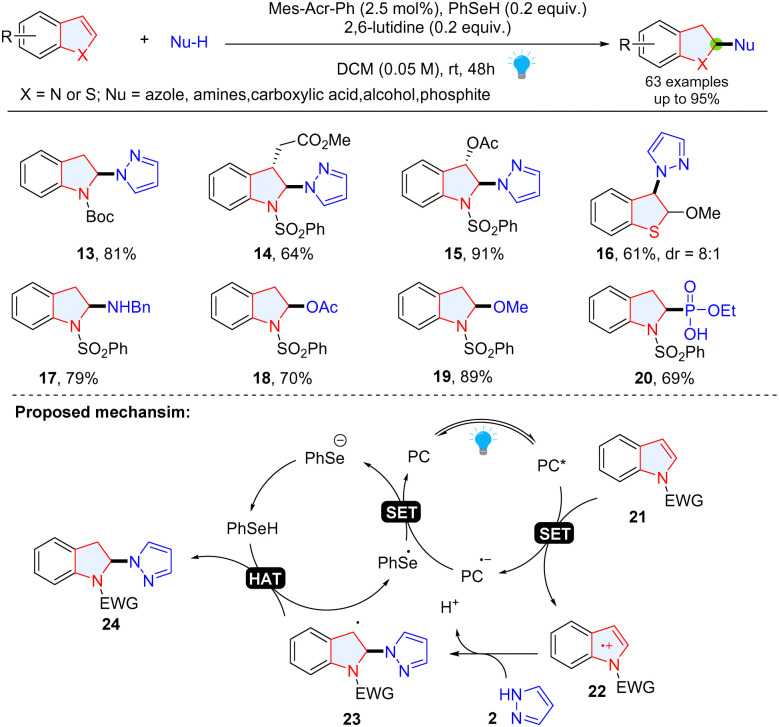
Umpolung strategy enabled dearomative functionalization of indoles/thiophenes.

Wang and coauthors also achieved the selective dearomatization of indole/benzothiophene *via* photoredox catalysis by selective oxidation of electron rich heterocyclic rings (Scheme 5).^33^ The radical cations 22 generated from photoredox mediated SET oxidation of indoles 21 reacts with nucleophiles and break the aromatic structure to form a benzylic carbon-cantered radical 23, which undergoes an HAT process, giving indolines 24. A variety of nucleophiles including azole, amine, carboxylic acid, alcohol and phosphite have been successfully employed in this reaction.

#### 2.1.2 Photoexcitation enabled dearomative hydrofunctionalization of bicyclic azoarenes

**Scheme 6:**
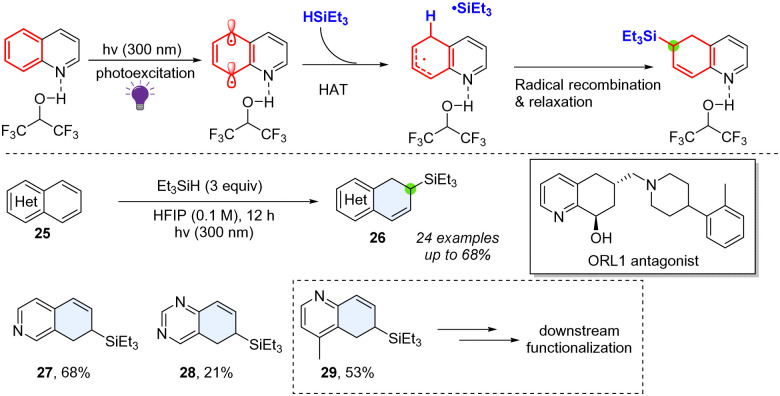
Direct photoactivated hydrosilyation of bicyclic azoarenes.

In 2023, Qin and co-workers reported a method for selective hydrosilylation or reduction on the phenyl site of (iso)quinolines (Scheme 6).^34^ The regioselectivity of the hydrosilylation reaction is substrate- and substituent-dependent, possibly due to the more electron-deficient radicals being photochemically generated at the 5-position of quinoline or 8-position of isoquinoline. The arenes of fused heterocyclic (iso)quinolines and quinazolines were selectively dearomatized. The produced allylsilane is a useful synthetic handle for further synthetic elaboration. This method could also be utilized in the synthesis of the opioid receptor-like 1 (ORL 1) antagonist (Scheme 6). Mechanistically, the key reactive diradical synthon was leveraged through UV-light and Brønsted acid coactivation. The presence of silanes facilitates the hydrogen atom and subsequent radical recombination, furnishing the desired products 26–29.

#### 2.1.3 Photoredox catalysed radical addition or radical–radical coupling for dearomative functionalization of five-membered ring fused heteroarenes

**Scheme 7:**
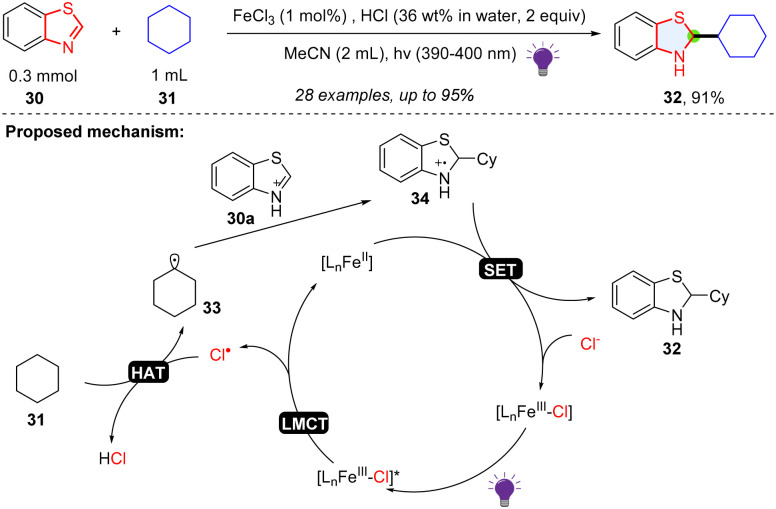
LMCT enabled radical addition to benzothiazole.

The five membered-ring in benzothiazole has an imine-like electrophilic property, which is proved to be a good radical trapper. Li and coworkers described a way of synthesizing benzothiazoline *via* photoredox catalysed dearomative alkylation of benzothiazoles (Scheme 7).^35^ FeCl_3_ plays the role as an indirect HAT reagent, and FeCl_2_ serves as the reducing agent. Simple cyclic alkanes, cyclic ethers, primary and secondary alcohols were shown to be good alkyl radical precursors for the hydroalkylation of benzothiazole. In the mechanism, the HCl and FeCl_3_ formed the Fe(iii)–Cl complex, which could be excited by 390–400 nm light. After the ligand-to-metal charge transfer (LMCT) process, a chlorine radical was generated and abstracted a hydrogen atom from cyclohexane 31 to form the alkyl radical 33, which was added to the protonated benzothiazole 30a and afforded radical cation intermediate 34. Subsequently, FeCl_2_ reduces the radical cation intermediate using a SET process, providing the target product 32 and closing the catalytic cycle.

**Scheme 8:**
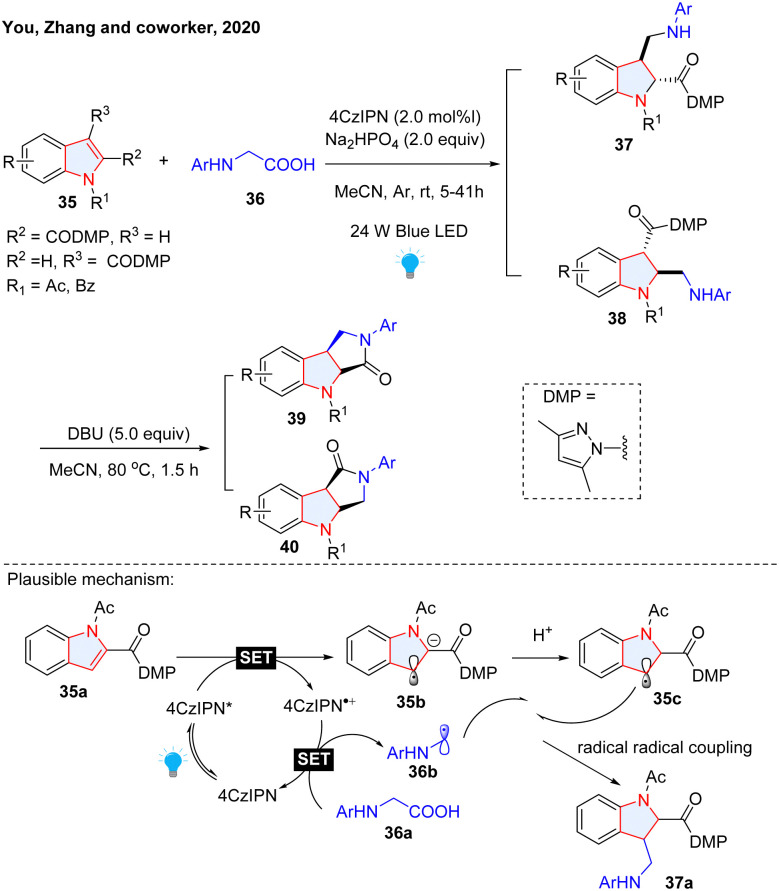
Radical–radical coupling for dearomatization of electron deficient indoles.

**Scheme 9:**
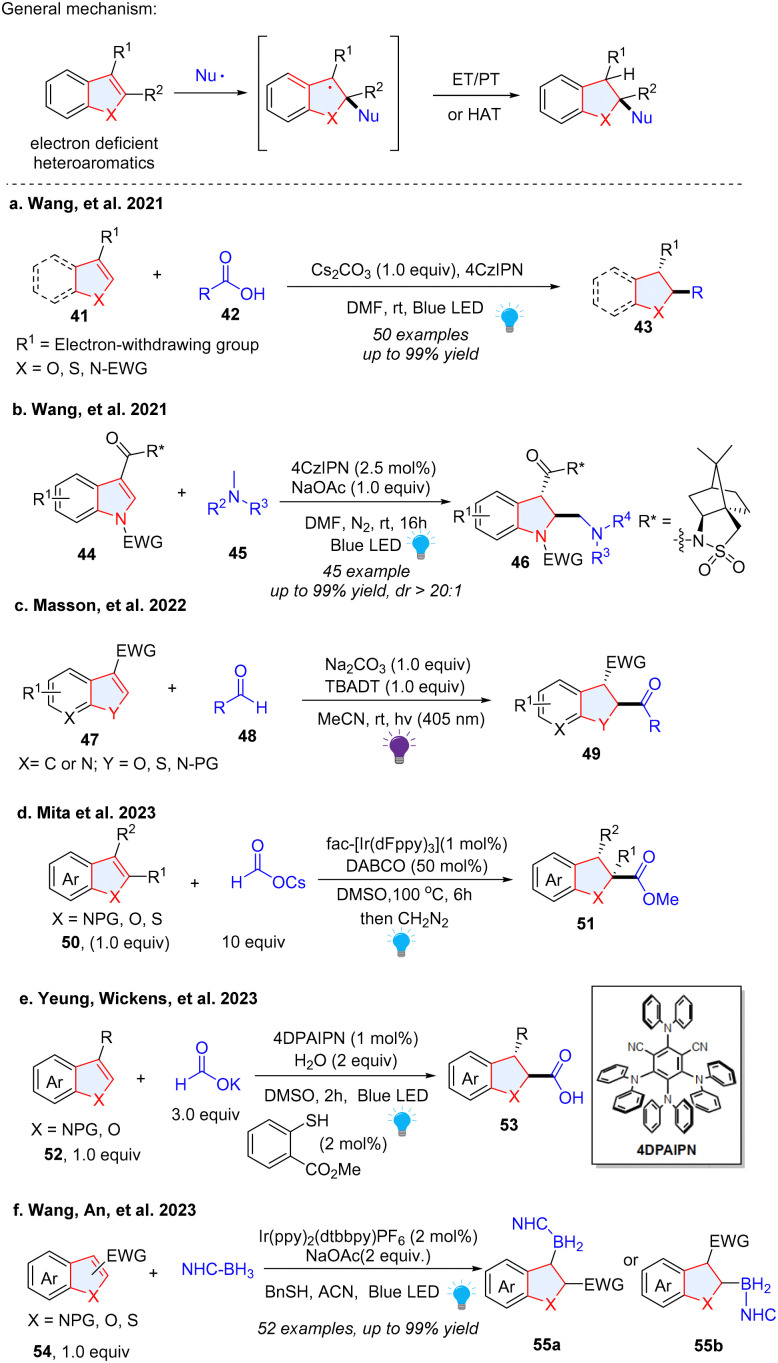
Nucleophilic radical addition to electron deficient indoles/benzothiopenes/benzofurans.

The nucleophilic reactivity of unsubstituted indoles possessing enamine-like electron-rich heteroarenes has been comprehensively investigated.^36^ Interestingly, when equipped with electron-withdrawing group(s) on the pyrrole ring of indole, its electron property is inverted to electron-deficient, rendering the electron transfer as well as nucleophilic radical addition feasible. For instance, Zhang and co-workers reported a photoredox catalysed radical-radical coupling protocol for dearomatization of such electron-deficient-indoles (EDIs, Scheme 8).^37^ The EDIs were dearomatized by alkyl radicals generated from photocatalyzed decarboxylation of glycine derivatives 36. Followed by DBU-mediated lactamization, lactam-fused indolines 39–40 were obtained in good to excellent yields. In the proposed mechanism, the indole was first reduced by photo-activated 1,2,3,5-tetrakis(carbazol-9-yl)-4,6-dicyanobenzene (4CZIPN) through a SET process, followed by protonation to deliver benzyl radical. 4CzIPN˙^+^ oxidized glycine to regenerate 4CzIPN and α-amino radical was delivered *via* releasing CO_2_. Eventually, cross-coupling of the two radical species formed the desired product. Our group reported similar reactions and the substrate scope was expanded to pyrroles and benzo(thio)furans (Scheme 9a).^38^ We proposed that this dearomatization is the result of nucleophilic radical conjugation. Considering the highly reactive radical species, stereoselective radical addition to EDIs is quite challenging. We successfully accomplished the asymmetric photocatalytic dearomatization of EDIs with neutral radicals generated from tertiary amines (Scheme 9b).^39^ Importantly, the incorporation of Oppolzer camphorsultam chiral auxiliary renders the radicals able to asymmetrically attack the planar structure of indole, generating indoline products with a single configuration. In addition to alkyl radicals, acyl radicals generated from aldehydes were also employed to dearomatize EDIs by Masson's group (Scheme 9c).^40^ In this protocol, tetra-*n*-butylammonium decatungstate (TBADT) plays the role of abstracting a hydrogen atom from aldehyde to form nucleophilic acyl radicals. The radical anion of CO_2_ (CO_2_˙^−^) is a strongly nucleophilic radical species, which has been employed in radical addition with electron-deficient/rich alkenes for the synthesis of aliphatic carboxylic acid.^41,42^ This highly reactive radical found its application in dearomative carboxylation of heteroarenes. The Mita group took advantage of CO_2_˙^−^, which was generated from cesium formate under photoredox/hydrogen atom transfer (HAT) catalysis, to dearomatize indole, benzofuran, benzothiophene and naphthalene (Scheme 9d).^43^ However, this method is limited by the low reaction efficiency and high reaction temperature (100 °C). Shortly, Yeung, Wickens, and co-workers reported a more efficient and milder strategy to realize the dearomative hydrocarboxylation of indoles and related heterocycles (Scheme 9e).^44^ Potassium formate was employed as the radical precursor for the generation of CO_2_˙^−^, which was added to the C-2 position of indole. Notably, the presence of thiol as a HAT catalyst greatly improved its efficiency. The transformation features mild reaction conditions, insensitive to both air and moisture, and it was readily amenable to high-throughput experimentation (HTE), which could be used to build libraries of structurally diverse 3D molecules. Other than the carbon-centred nucleophilic radical, the boron radical was also reported to participate in dearomative functionalization of indole, benzothiophene, and benzofurans (Scheme 9f).^45^

**Scheme 10:**
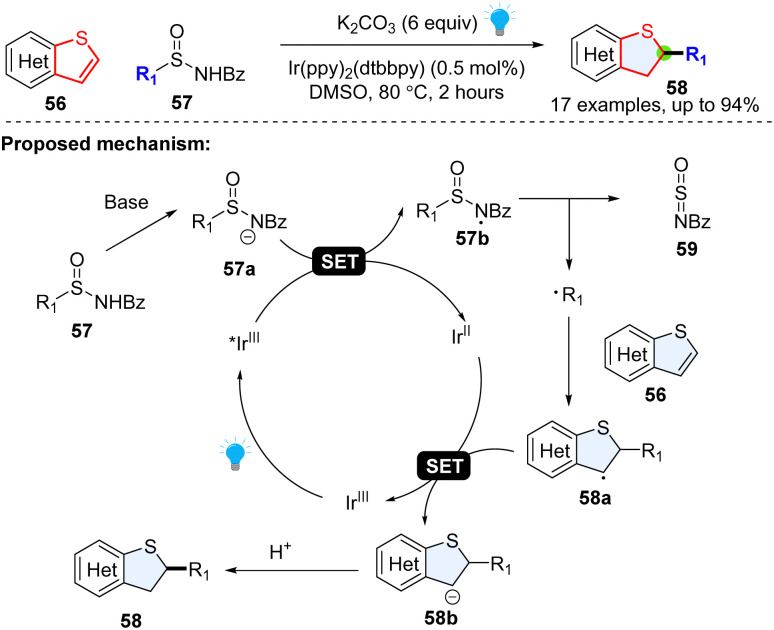
Desulfuration enabled nucleophilic radical addition to electron rich benzothiophenes.

Although the thiophene ring is electron-rich, it was also reported to behave like a radical acceptor. For example, in 2018, Qin and colleagues achieved a regiospecific hydroalkylation of benzothiophenes enabled by photoredox catalysed desulfuration (Scheme 10).^46^ It was serendipitously found that alkyl radical precursors *N*-acyl alkyl-sulfinamides could undergo a Giese-type reaction at the 2-position of the heteroaromatic. Mechanistically, the *N*-acyl-alkyl-sulfinamide 57 was deprotonated to deliver the anion 57a, which was readily oxidized by photoexcited *Ir^III^ to generate nitrogen centred-radical 57b. The fragmentation of nitrogen centred radical 57b yielded *N*-sulfinylbenzamide 59 and the corresponding alkyl radical R^1^˙. The formed alkyl radical attacks the thiophene 56, generating the stable benzylic radical intermediate 58a, followed by the reductive SET process and protonation, producing the desired product 58.

**Scheme 11:**
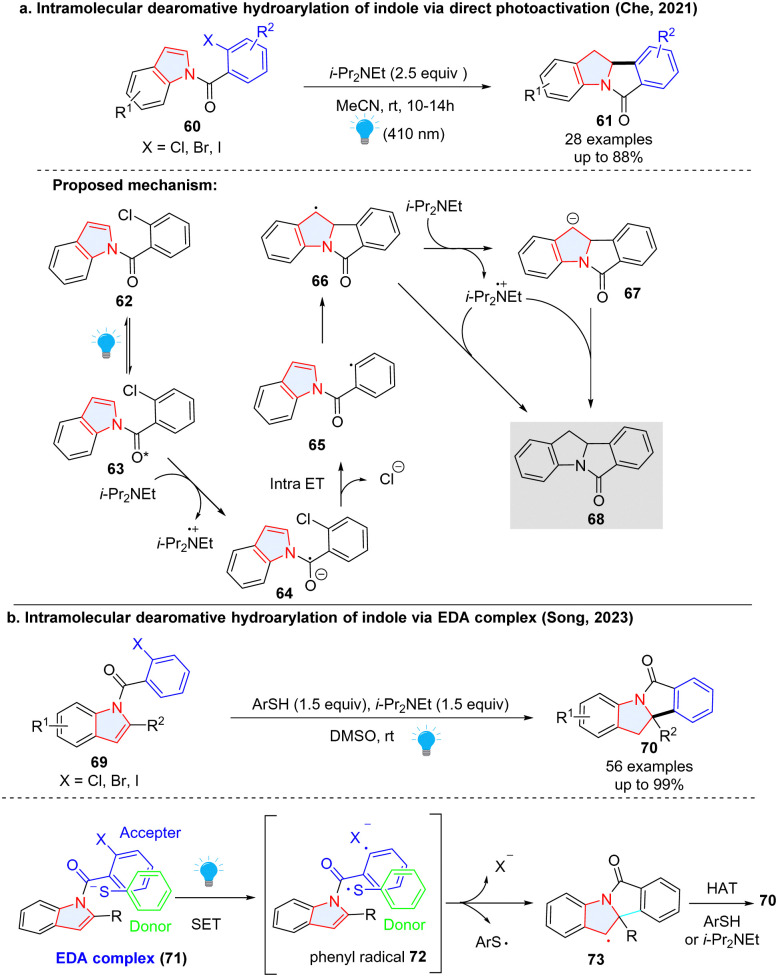
Intramolecular radical addition to electron deficient indoles.

In addition to the aforementioned intermolecular dearomative radical addition to heterocycles, the intramolecular version was also reported to be an efficient way to construct polycyclic indolines. In 2021, Che and colleagues described a photo-induced reductive Heck cyclization of indoles for the efficient preparation of polycyclic indolinyl compounds from *N*-(2-chlorobenzoyl) indoles without using a photocatalyst (Scheme 11a).^47^ Upon irradiation, indole 63 in the long-lived excited state was reduced by i-Pr_2_NEt, forming the radical anion 64. The C(sp^2^)–Cl bond was intramolecularly activated, fragmenting into a phenyl radical 65 and chloride ion. Intramolecular radical addition led to benzyl radical 66. Finally, the HAT with i-Pr_2_NEt˙^+^ or further reduction of 66 to carbanion 67 followed by protonation gave the reductive Heck cyclization product 68.

Two years later, Song and coworkers disclosed a similar dearomative cyclization of indoles (Scheme 11b).^48^ Different from Che's work, thiophenol is necessary for the indoles with substitutions at the C-2 position. Mechanistically, the electron donor–acceptor (EDA) complex 71 formed between indole and thiophenol was activated under the irradiation to generate phenyl radical and thiophenol radical *via* a SET. The intramolecular dearomatization approach gave benzyl radical 73 which obtained a hydrogen atom from thiophenol or i-Pr_2_NEt to deliver the final product 70.

#### 2.1.4 Dearomative radical 5-exo-trig spirocyclization of electro-rich thiophene/furan/indole

**Scheme 12:**
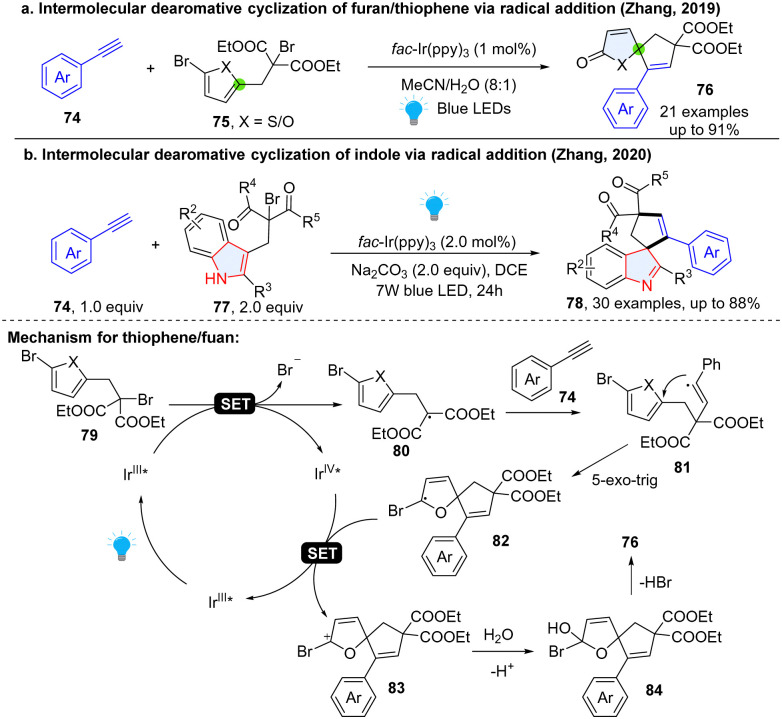
Dearomative radical spirocyclization of electron rich thiophenes/furans/indoles.

Spirocyclic motifs broadly exist in bioactive molecules and natural products.^49,50^ Therefore, seeking simple and efficient methods for the construction of spiro-scaffolds is of great interest in synthesis and drug discovery.^51^ Recent years witnessed great advancement for photochemical dearomative spirocyclization of heteroaromatics. Interestingly, most of reported examples involved a Baldwin favoured 5-exo-trig or 4-exo-trig ring cyclization process. For instance, in 2019, Zhang and coworkers^52^ developed an intermolecular, photoredox catalysed dearomative spirocyclization of thiophens/furans to synthesize useful 1-oxaspiro[4.4]nona-3,6-dien-2-one 76 (Scheme 12a), featured in many natural products such as (−)-securinine, secosyrin, and hyperolactone. This method employed alkynes 74 and 2-bromo-1,3-dicarbonyl compound 75 as reactants. Once irradiated by visible light, the excited *Ir^III^ species reduce the thiophene/furan substrate 79 after a SET process, subsequently generating alkyl radical intermediate 80. The formed radical intermediate 80 undergoes a rapid addition to ethynylbenzene to afford an electrophilic vinyl radical intermediate 81. A thermodynamically favourable 5-exo-trig radical cyclization takes place on the thiophene/furan, generating a key radical 82, which is oxidized by Ir^IV^ to form cation intermediate 83 and complete the photoredox catalytic cycle. After water involved hydrolysis and elimination, desired product 76 was delivered. Later, the same group applied a similar reaction to dearomatize indoles for the synthesis of bioactive spiroindolenines 78 (Scheme 12b), the common substructures in alkaloid natural products.^53^

**Scheme 13:**
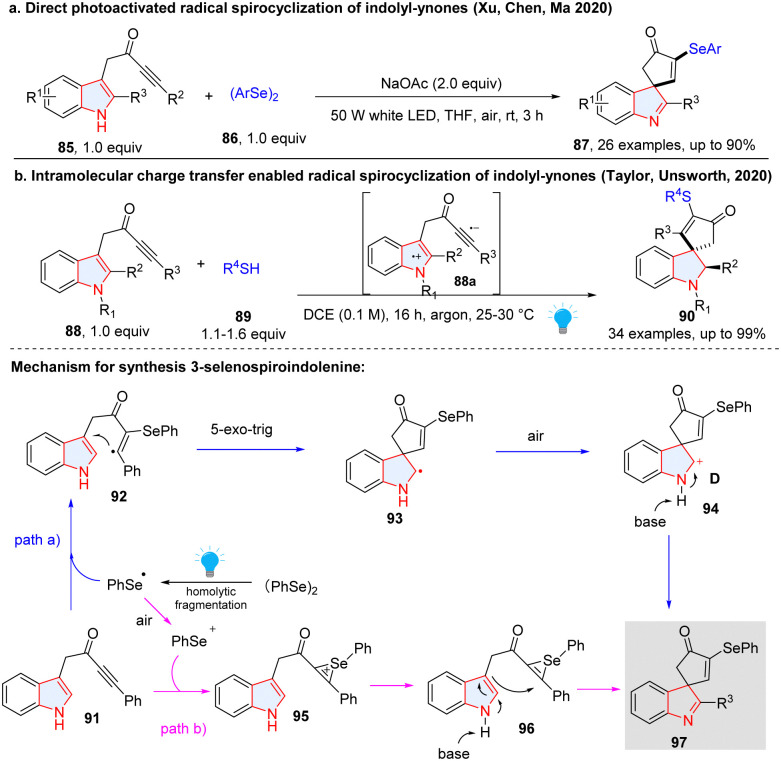
Dearomative radical spirocyclization of indoles for the synthesis of seleno/thiospiroindolenines.

Meanwhile, Xu, Chen, Ma and co-workers reported a photochemical method for the construction of 3-selenospiroindolenines through spirocyclization of indolyl-ynones under air without a photocatalyst (Scheme 13a).^54^ Interestingly, they found the reaction may proceed through both radical and ionic pathways. In the radical pathway (path a), PhSe˙ generated from homo-cleavage of diphenyl diselenide was added to indolyl-ynone 91, producing radical 92. 5-exo-trig ring closure with the indole at its 3-position would occur, forming a spirocyclic radical intermediate 93. Oxidation of 93 in the air followed by dehydrogenation in the base condition afforded product 97. In the ionic pathway (path b), PhSe˙ was oxidized to PhSe^+^ in the air, which then reacted with the alkyne group of indolylynone 91 resulting in the formation of selenium ion 95. Then, 95 was cyclized at C3 of indole to give the final compound 97 with the aid of a base. Similarly, Unsworth and Taylor disclosed a visible-light-induced radical spirocyclization of indoyl ynones for the synthesis of thiospiroindolenines (Scheme 13b).^55^ Distinctly, this reaction was initiated by the visible-light promoted intramolecular charge transfer of indolyl ynones 88, furnishing the indolyl radical cation and alkynylradical anion 88a, which interacted with thiol 89 and generated a thiyl radical. The subsequent radical addition and spirocyclization have the same mechanism as shown in Scheme 13a. The anaerobic conditions lead to the formation of the spirocyclic indoline (90).

**Scheme 14:**
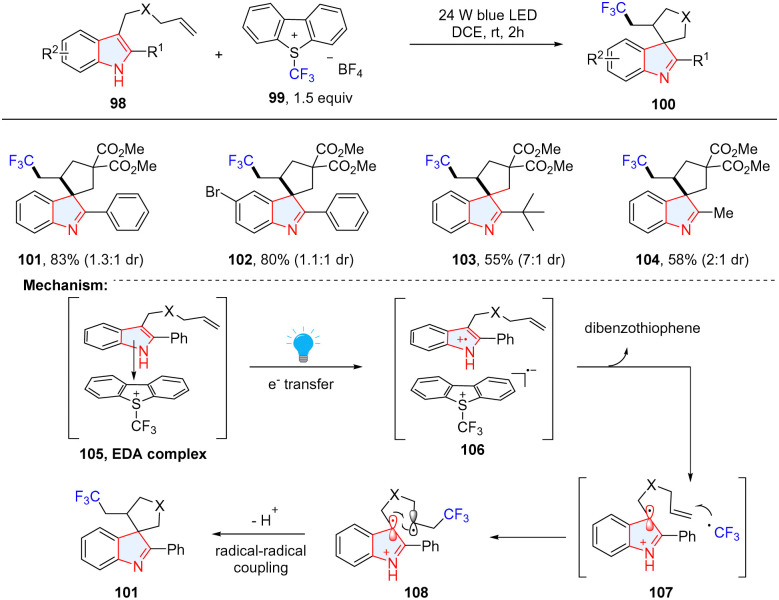
EDA complex induced dearomative radical spirocyclization of electron rich indoles.

In addition to the vinyl radical induced dearomative spirocyclization, alkyl radical has a similar reactive behaviour. By taking advantage of the electron donor–acceptor complex between indole derivatives 98 and Umemoto's reagent 99, You, Zhang and coworkers achieved a dearomative spirocyclization of indole derivatives *via* visible-light-promoted cascade alkene trifluoromethylation (Scheme 14).^56^ The combination of indole and Umemoto's reagent generated the transient complex 105, which underwent a SET from donor to acceptor upon the irradiation of blue light (106). The CF_3_ radical generated through S–CF_3_ bond cleavage proceeds *via* an addition to terminal alkene (107) to afford the intermediate 108. Ultimately, radical–radical recombination occurs to provide the desired product 101 after deprotonation.

#### 2.1.5 EnT promoted dearomative 4-exo-trig spirocyclization of indole through radical–radical cross coupling

**Scheme 15:**
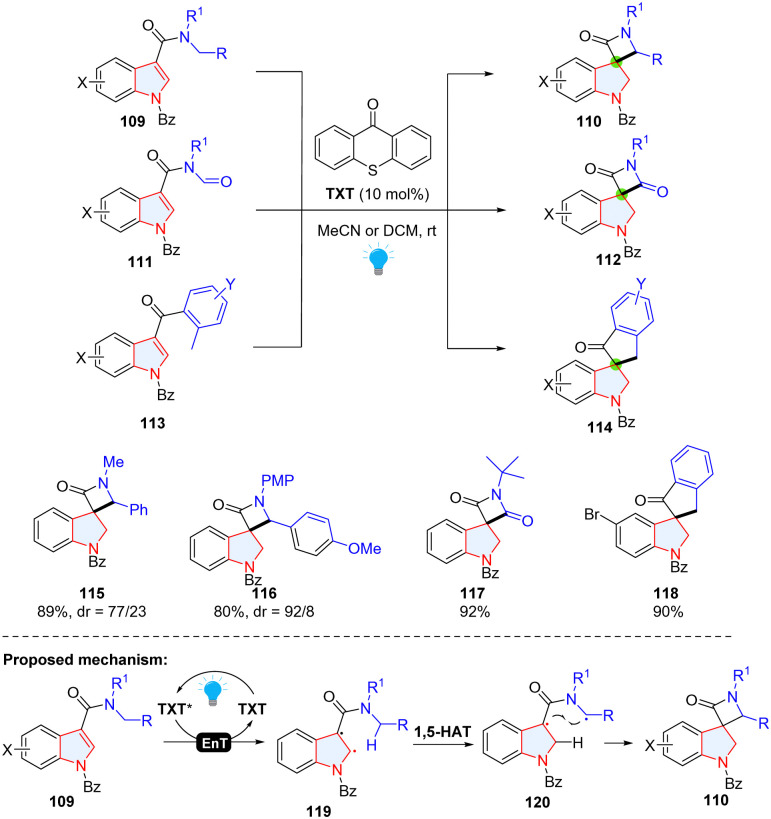
EnT promoted dearomative 4-exo-trig spirocyclization of indoles *via* intramolecular radical–radical cross coupling.

Indoles with electron-withdrawing groups becoming electron-deficient indoles (EDIs) have been recently used in the Giese-type dearomatization (see section 2.1.3). Additionally, the C2=C3 in an EDI can be activated to form a diradical intermediate which can participate in [2+N] cycloaddition.^57^ Recently, Bach's group achieved an energy transfer (EnT) catalysed dearomative hydrogen atom abstraction/cyclization cascade of EDIs for the synthesis of spirocyclic indolines (Scheme 15).^58^ Based on the control experiments and previous reports, the authors proposed that once thioxanthen-9-one (TXT) was activated by visible light to form TXT*, the energy was transferred from TXT* to EDI (109), generating diradical intermediate 119. Through intramolecular 1,5-HAT, C2 in 119 abstracted a hydrogen atom to produce diradical intermediate 120, which underwent ring closure through radial-radical coupling to deliver product 110.

### 2.2 Multiple-point dearomative modifications of heteroaromatics

Compared to single point modifications, the multiple point dearomative functionalization of heteroaromatics can incorporate more complexity and functionality into the newly formed 3D structures during the dearomatization process. Due to the issues of chemo-, regio-, and stereoselectivity, this type of reaction remains quite challenging. Recently, several groups have made great contributions to this field by elaborating on a combination of photocatalyzed radical approaches and traditional ionic pathways (*e.g.* biocatalysis, organocatalysis, transition-metal catalysis).

#### 2.2.1 Direct visible-light activated arenophiles for multiple dearomative functionalization

**Scheme 16:**
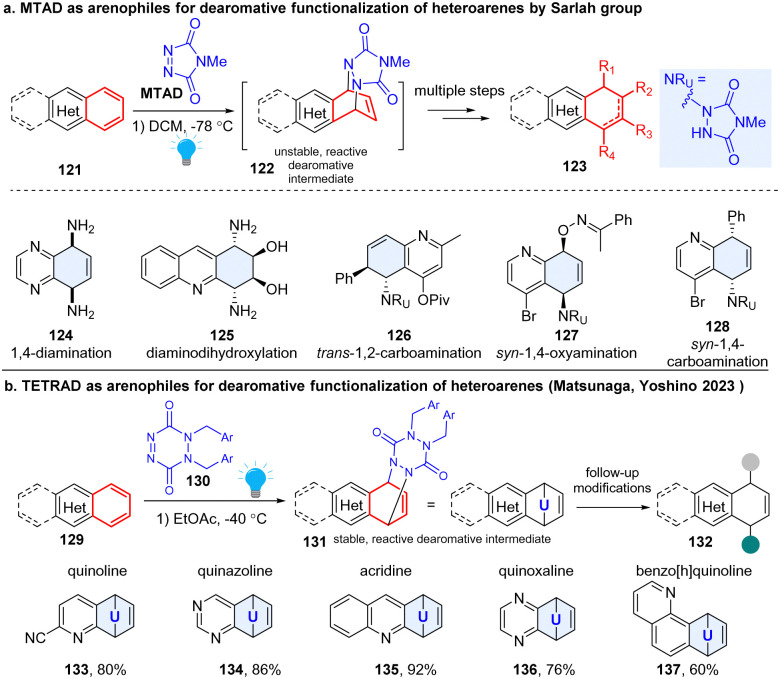
Photoactive arenophile induced multiple dearomative functionalization of heteroarenes.

Sarlah pioneered the field and developed a robust platform for dearomatization and multiple functionalization of diverse non-activated arenes (Scheme 16a). This strategy employed a photoactivable 4-methyl-1,2,4-trazoline-3,5-dione (MTAD), termed as ‘arenophiles’, which can formally dearomatize the simple heteroarenes 121 in a [4+2] fashion by forming a cycloadduct 122 chemoselectively with electron rich arenes.^59^ Subsequently, the reactive cyclized intermediates would undergo retrocycloaddition or fragmentation, delivering dearomatized products 123 substituted by diverse functional groups. This approach could simultaneously disrupt aromaticity, insert functionality, and produce stereogenic centres. For example, they achieved diverse transformations with inert arene moieties of the polycyclic heteroaromatics, such as *syn*-1,4-diamination (124),^60^ diaminodihydroxylation (125),^59^*trans*-1,2-carboamination (126),^61^*syn*-1,4-oxyamination (127),^62^ and *syn*-1,4-carboamination (128),^63^*etc.* Importantly, many natural products such as MK7607 or bioactive drugs (phomentrioloxin) were efficiently synthesized using this approach.^59^ It should be noted that the arene-arenophile cycloadduct is thermally unstable. The isolation and further characterization of cyclized products are difficult, and the subsequent functionalization needs to be carried out at low temperature, which potentially limits its application. In 2023, the Matsunaga and Yoshino group reported a new six-membered arenophile, 1,2-dihydro-1,2,4,5-tetrazine-3,6-diones (TETRAD, 130), which can also undergo visible-light-induced [4+2] cycloaddition reaction with a number of nonactivated polycyclic heteroarenes (Scheme 16b), including quinoline (133), quinazoline (134), acridine (135), quinoxaline (136), and benzo[h]quinoline (137).^64^ The cyclized adducts 131 are stable for isolation and can be characterized using single-crystal X-ray diffraction, which validates a twisted-boat-like conformation for the *p*-urazine moiety. Density functional theory calculations revealed that the benzene-TETRAD adduct 131 proceeds *via* asynchronous cleavage of two C–N bonds with a concomitant large conformational change of the *p*-urazine moiety from the adduct to the transition state, while the benzene-MTAD undergoes retro-cycloaddition through a synchronous mechanism. As expected, the isolated adducts can be utilized for further synthetic transformations, such as palladium-catalysed *syn*-1,4-carboamination, copper-catalysed *trans*-1,2-carboamination, and *syn*-1,4-diamination, *etc*.

**Scheme 17:**
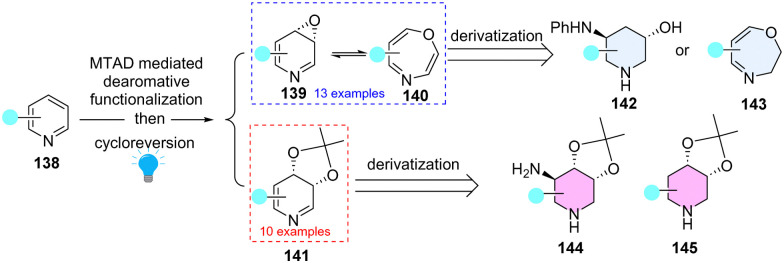
Arenophile mediated dearomative multi-functionalization of pyridines.

Dearomatization of pyridines can efficiently access piperidines motifs, abundant in FDA-approved drugs and natural products. However, these dearomatization approaches mainly relied on the hydrogenation of pyridines or the addition of *C*-nucleophiles to activated pyridinium salts. Recently, Sarlah and coworkers^65^ elegantly demonstrated photochemical dearomative multi-functionalization of pyridines by direct introduction of heteroatom functionalities (O/N) by combination of arenophile chemistry and olefin oxidations (Scheme 17). Importantly, the derived dihydropyridine *cis*-diols (141) and pyridine oxides (139) are amenable to deliver downstream functionalizations (142–145), which provide an efficient way to construct high-value sp^3^ enriched heterocycles.

As shown above, visible-light-activated arenophiles induced reactions involve multiple-point dearomative functionalization with the saturated six-membered scaffold unchanged. Recently, based on the arenophile chemistry, Sarlah and colleagues successfully achieved dearomative skeletal editing by inserting one ‘oxygen’ or ‘carbon’ atom into the dearomatized arenes, which will be discussed in the third part of the review.

#### 2.2.2 Visible-light driven dearomative triple elementalization of quinolines

**Scheme 18:**
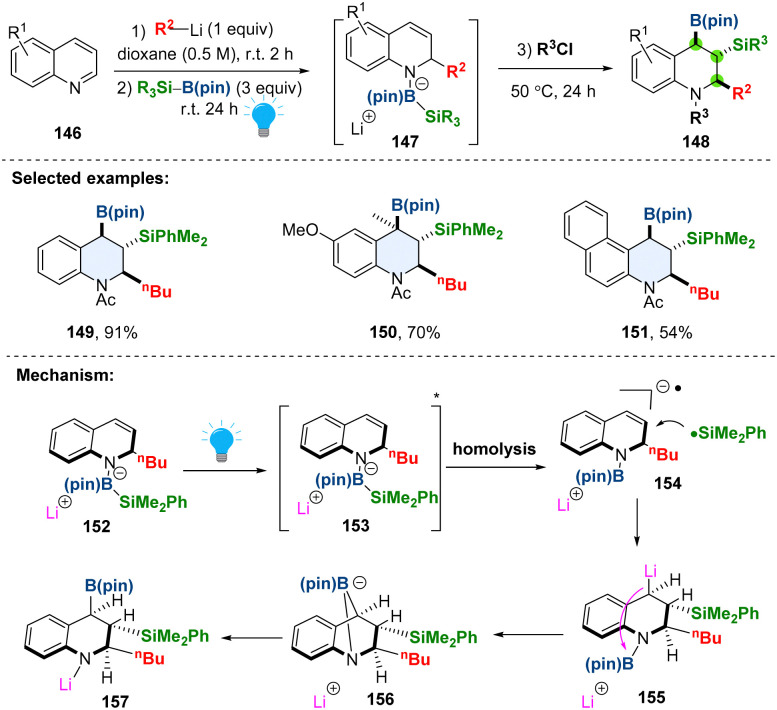
Direct visible light promoted dearomative triple elementalization of quinolines.

Dearomative borylation or silylation of heteroaromatics in a stereoselective fashion enables access to aliphatic nitrogen heterocycles bearing multiple boryl or silyl groups, which are versatile handles in synthesis.^66,67^ The currently available methods are limited to either dearomative hydroboration or hydrosilylation, the silaboration in one protocol remains elusive. Besides, the visible-light promoted dearomative tri-functionalization is even rarer. In 2023, Tanaka and coworkers developed a chemo-, regio-, and stereo-selective dearomative triple elementalization (carbo-sila-boration) of quinolines by combining the organolithium addition and photo-boosted silaboration (Scheme 18).^68^ In the proposed reaction pathway, the borate intermediate 152 could be selectively excited by photo-irradiation to a singlet excited state 153, which would undergo homolysis of the Si–B bond, generating both silyl radical and radical anion species 154. The radical coupling produces the silylmetalated intermediate 155, followed by boryl group transfer from the nitrogen atom to the carbon atom through bicyclo intermediate 156, affording the desired product 157. The steric hindrance in the intermediate dictates the anti-conformation of boryl and silyl groups. As demonstrated, the synthesized carbo-sila-borated tetrahydroquinolines can be converted into another versatile functionality including carbooxy-silylation, carbosila-deuteration, dicarbon-silylation, and dicarbon-oxidation products.

#### 2.2.3 Cooperative photoredox and enzyme/organo/metal co-catalyzed dearomative difunctionalization of indole/benzothiophene/benzofurans

**Scheme 19:**
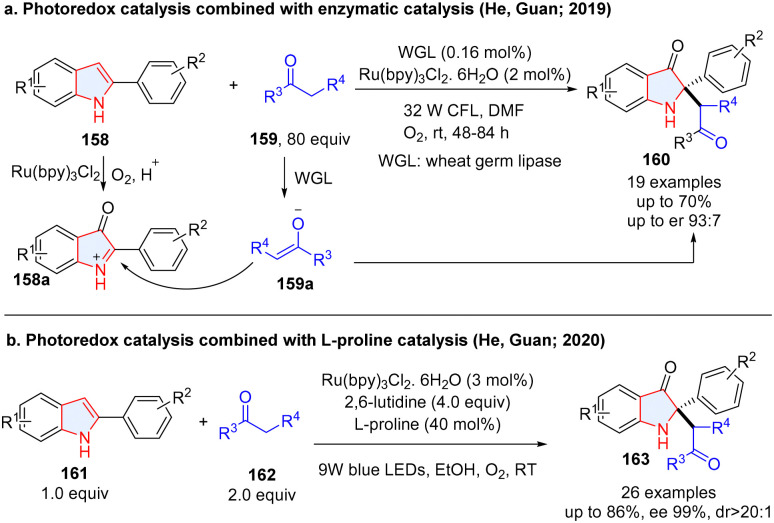
(a) Photoredox catalysis with enzymatic catalysis for asymmetric dearomative 2,3-difunctionalization of indoles; (b) photoredox catalysis coupling with proline catalysis for enantioselective dearomative 2,3-difunctionalization of indoles.

To achieve the multiple dearomative functionalization of heteroarenes, photoredox catalysis has also been combined with ionic catalytic methodologies including enzymatic catalysis, organocatalysis (chiral secondary amine, N-heteroaromatic carbene, chiral phosphoric acid, *etc*), and transition-metal catalysis. In this section, we summarize the achievements. Although an enzymatic dearomatization strategy proved to be a powerful strategy for enantioselectively building up 3D molecular complexity,^69–71^ cooperative photoredox/biocatalytic dearomatization is quite rare. In 2019, He, Guan and co-workers described the fusion of photoredox and enzymatic catalysis for the asymmetric synthesis of 2,2-disubstituted indol-3-ones from 2-arylindoles 158 (Scheme 19a).^72^ In the process, 2-arylindoles 158 were oxidized to 2-arylindol-3-one 158a under photoredox conditions by O_2_, followed by enantioselective alkylation with ketones catalysed by wheat germ lipase (WGL). However, this reaction is limited by low yields and moderate enantioselectivity. Later, they improved the reaction efficiency and enantioselectivity by merging photoredox catalysis and l-/d-proline amniocatalysis.^73^ The 2-arylindol-3-one was first generated *via* photocatalysis, followed by sequent alkylation with ketone catalysed by l-proline to give products (163) with higher enantioselectivities (Scheme 19b).

**Scheme 20:**
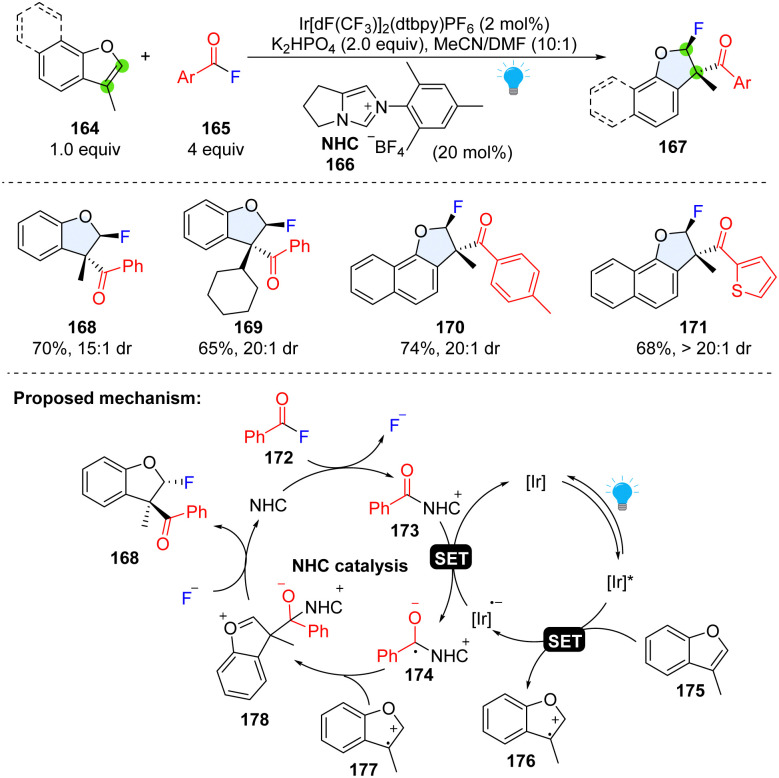
Cooperative photoredox catalysis and NHC catalysis for dearomative 2,3-difunctionalization of benzofurans.

The double bond in the five member-ring of benzofurans exhibits a similar reactivity to indole, which can undergo the SET oxidation process, generating a reactive radical cation in the furan ring. Although the arene radical cations are extensively studied by combination with nucleophile, their trapping by a carbon centred radical is underexplored. By manipulation of the reactive arene radical cation intermediate and cooperative NHC/photoredox catalysis generated-radical anion, Studer and coworkers achieved the di-functionalization of benzofurans using aroyl fluorides and anhydride as bifunctional reagents (Scheme 20).^74^ Diverse 2,3-dihydrobenzofuran building blocks (167), core motifs appearing in biologically active compounds, were synthesized with moderate to good yield and high diastereoselectivity (168–171). A mechanism was proposed that upon visible light irradiation, 3-methylbenzofuran 175 is readily oxidized to radical cation 176 by *Ir(iii). Meanwhile, the NHC catalyst reacts with aroyl fluoride 172 to deliver the acyl azolium ion 173, which is reduced by Ir(ii) with the concurrent formation of persistent ketyl radical 174. The radical–radical coupling between radical cation 176 and ketyl radical 174 results in an oxocarbenium ion 178. The bulky alcoholate moiety renders the F^−^ anion nucleophilic attack favourable in the *trans*-position, and subsequent NHC fragmentation yields difunctionalized product 168 to complete the NHC-catalysed cycle.

**Scheme 21:**
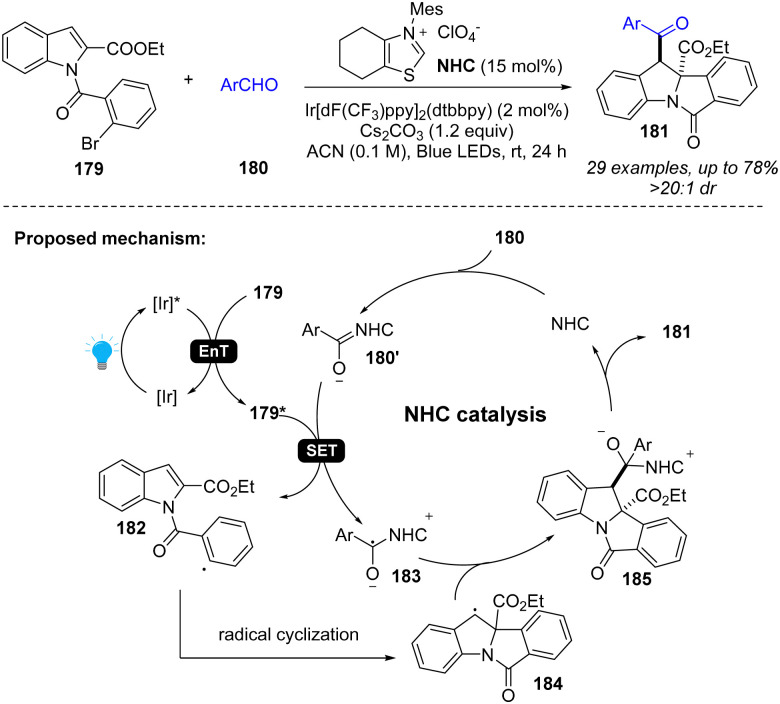
Cooperative EnT and NHC catalysis for dearomative 2,3-difunctionalization of indoles.

In addition to photoredox catalysis, energy transfer catalysis was also integrated into NHC catalysis for dearomatization of indoles (Scheme 21).^75^ Specifically, the photocatalyst and visible light promote the indole derivative 179 to an excited state 179*, which can oxidize the Breslow intermediate 180′, generating benzyl radical 183 and aryl radical 182. After radical cyclization, the radical cross-coupling between two benzyl radicals 183 and 184 forms the intermediate 185 and returns the NHC catalyst, providing desired difunctionalized indoline product 181.

**Scheme 22:**
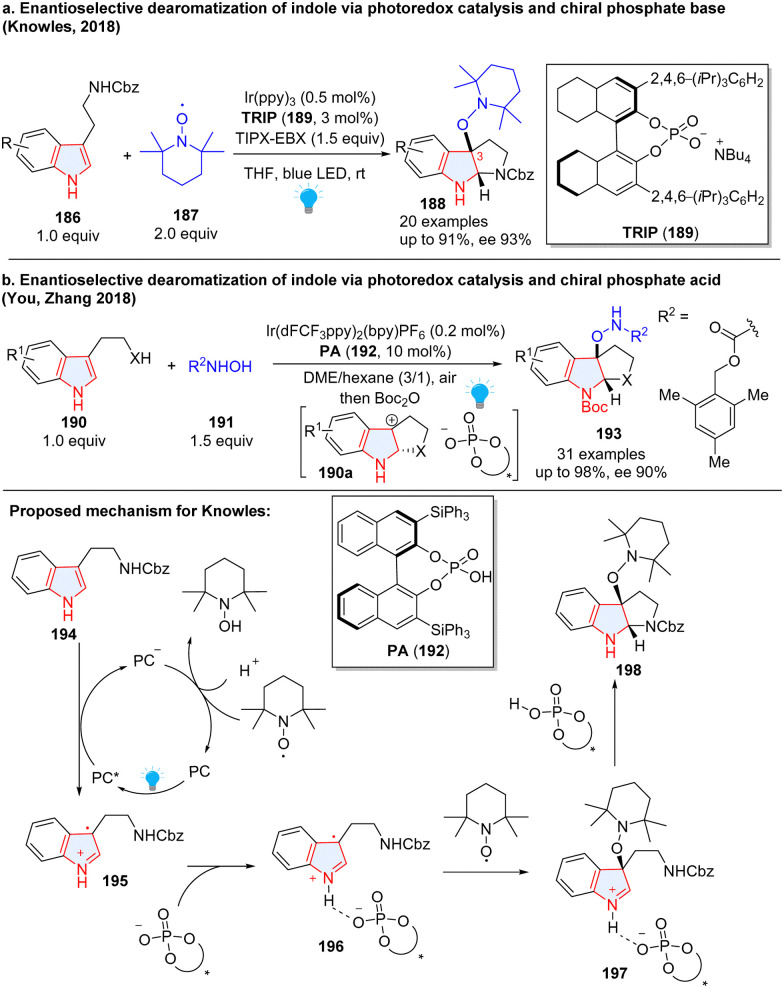
Cooperative photoredox catalysis and chiral phosphate acid/base catalysis for dearomative 2,3-difunctionalization of indoles.

As chiral phosphates have been widely applied in asymmetric catalysis, to realize the photocatalytic asymmetric dearomatization of indoles, the Knowles group elegantly utilized chiral phosphates to enantioselectively synthesize C3-substituted pyrroloindolines 188 from tryptamine derivatives 186 (Scheme 22a).^76^ In the reaction, tryptamine 186 was first oxidized by excited PC* to produce radical cation 195, which was stabilized by chiral phosphate anion 196 through hydrogen-bonding. The noncovalent open-shell complex 196 was intercepted by the stable nitroxyl radical 2,2,6,6-tetramethylpiperidine 1-oxyl (TEMPO), followed by intramolecular nucleophilic radical addition to form alkoxyamine-substituted pyrroloindoline 198 with high enantioselectivity. Furthermore, these enantioenriched products can be oxidized to generate transient carbocation intermediates that can be trapped by a wide range of nucleophiles including trifluoroborate nucleophiles, silyl nucleophiles, alcohols, anilines and sulfamates. This protocol was successfully applied to the enantioselective synthesis of natural product (–)-calycanthidine and (–)-psychotriasine. Later, a similar transformation was reported by Xia and coworkers.^77^ You, Zhang and co-workers reported a similar reaction.^78^ Instead of enantioselectively trapping radical cation 196 by TEMPO radical 187, they successfully employed *N*-hydroxycarbamates 191 as a nucleophile to intercept carbocation 190a, furnishing optically pure product 193 (Scheme 22b).

**Scheme 23:**
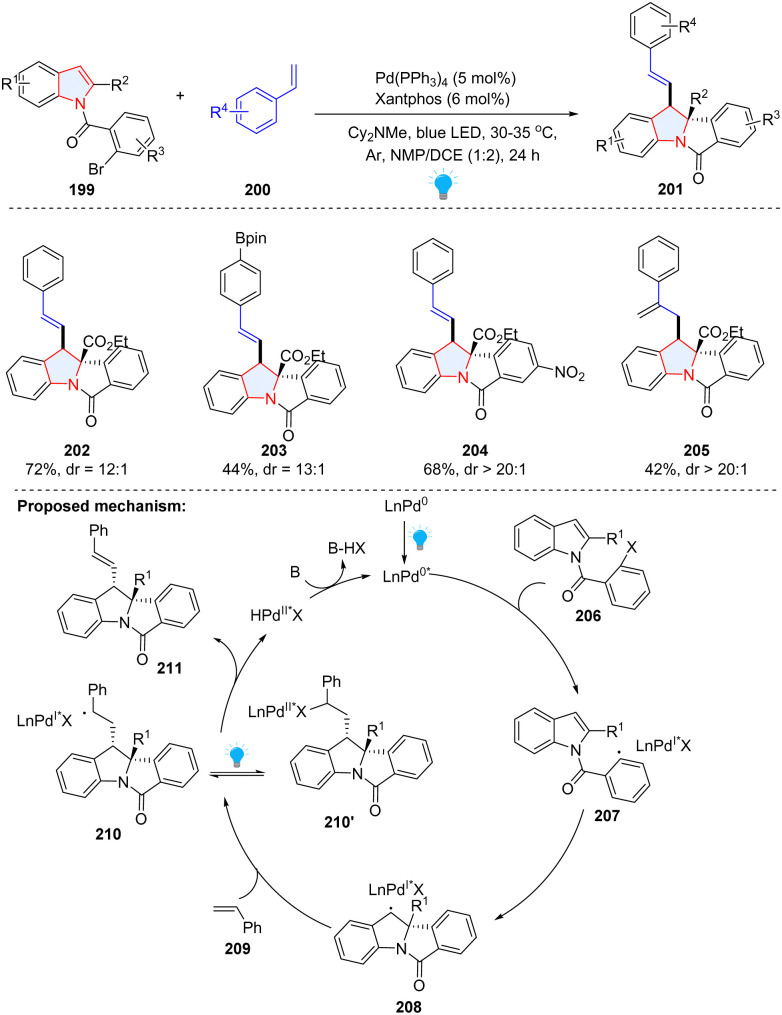
Visible-light activated palladium catalysed dearomative 2,3-difunctionalization of indoles.

Compared to conventional palladium catalysis, a visible-light-driven Pd-catalysed photoredox reaction can significantly lower the energy barrier under milder conditions. In 2022, the Sharma group presented a photochemical palladium-catalysed dearomatization of indoles by utilizing unactivated alkenes 200 and *N*-(2-bromobenzoyl)indoles 199 as starting materials (Scheme 23).^79^ In the proposed mechanism, the aryl bromide was reduced by photoexcited Pd(0) into aryl radical 207, followed by facile intramolecular radical addition to the C2–C3 of indole, affording benzyl radical 208. Subsequently, indole-palladium complex 208 was added to styrene 209, forming the hybrid alkyl Pd(i) radical species 210, which is in equilibrium with alkyl Pd(ii) species 210′ under irradiation. The β-H elimination of 210′ produced the final product 211 with concomitant regeneration of the Pd(0) species.

#### 2.2.4 Photoredox induced cascade radical conjugation for dearomative difunctionalization of heteroarenes

Cascade reactions represent an efficient strategy to incorporate multiple functionalities within one vessel operation. With the revival of photocatalysis, a series of photochemical cascade dearomative functionalization of five-membered ring fused heteroarenes (indole/benzothiophene/benzofuran) have been uncovered. In the transformations, the photoredox- or EnT-generated radical species usually attack the 5-member ring first, forming stable benzylic radical or anion, which then undergoes another different chemical transformation to install the second functionality on the dearomatized ring. Below, summarized examples are demonstrated in detail.

**Scheme 24:**
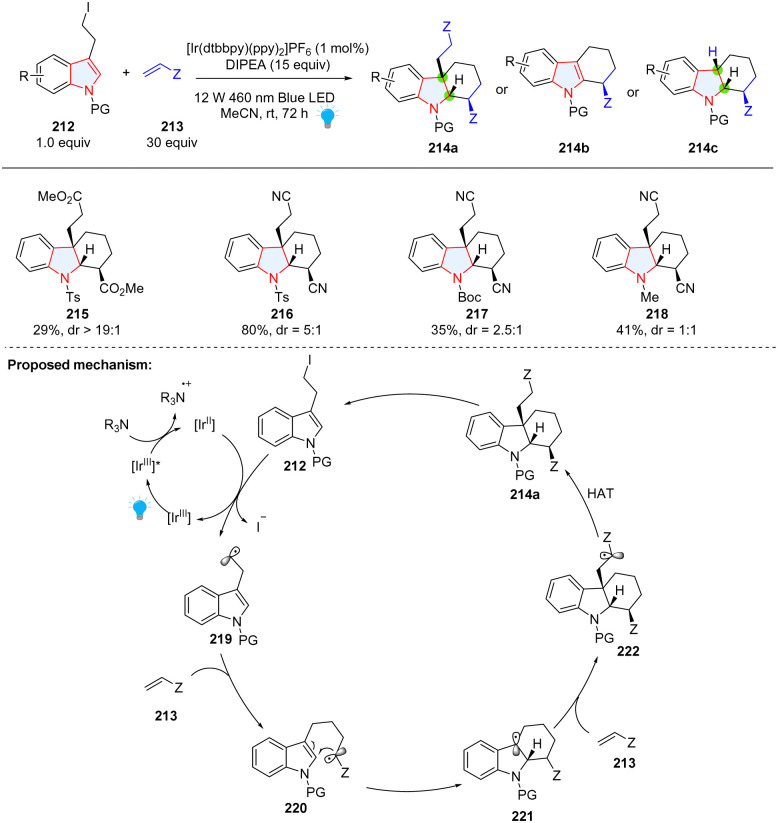
Photoredox catalyzed cascade dearomative 2,3-difunctionalization of indoles using alkenes as a trapper.

Intermolecular reaction followed by cyclization is a powerful strategy to construct polycyclic compounds. Starting from 3-(2-iodoethyl)indoles 212 and substituted alkenes 213, the Brasholz group reported the synthesis of highly functionalized hexahydro-1*H*-carbazoles 214a through a dearomative radical cyclization/1,4-addition cascade (Scheme 24).^80^ In their proposed mechanism, 3-(2-iodoethyl)indole 212 was reduced by [Ir^II^] to form nucleophilic radical 219, which was added to electron-deficient alkene 213 resulting in electrophilic radical 220. Radical cyclization of 220 generated a nucleophilic radical 221, which can be trapped by the second Michael acceptor 213. In this methodology, electron-deficient alkene was limited to acrylonitrile and a large amount (30 equiv.) was necessary, which was attributed to the formation of byproducts 214b and 214c.

**Scheme 25:**
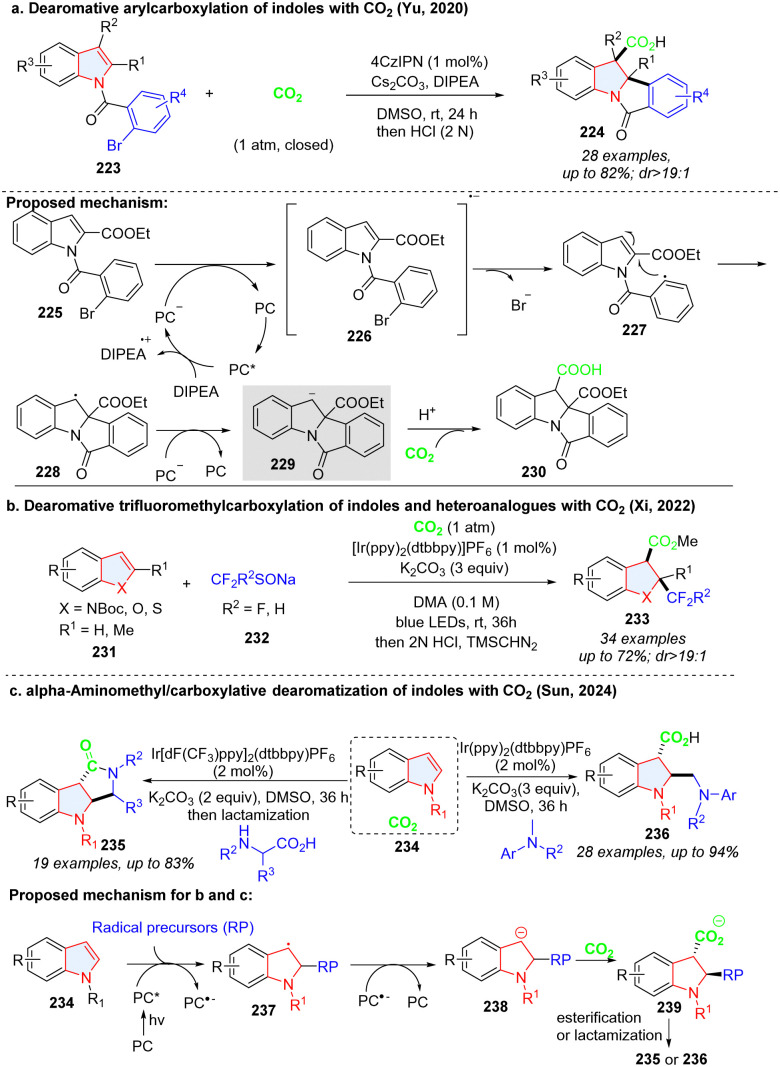
Photoredox mediated cascade dearomative 2,3difunctionalization of indoles utilizing CO_2_ as a trap.

Yu's group dexterously devised a photoredox catalysed net reductive reaction, in which CO_2_ was utilized to trap the stable benzylic anion intermediate 229, furnishing the indoline-3-carboxylic acids (Scheme 25a).^81^ Similar to Yu's work, Xi described the intermolecular dearomative trifluoromethylcarboxylation of indoles or heteroanalogues (benzothiophene, benzofuran) with CO_2_ and sodium trifluoromethanesulfinate 232 (Scheme 25b).^82^ The electrophilic radical CF_3_˙, which was generated from SET oxidation of sodium trifluoromethanesulfinate 232, reacted with electron-rich indole 231 to form benzyl radical 237, followed by reduction to the benzyl anion 238 by a photocatalyst. Similarly, the anion intermediate could be captured by CO_2_, and underwent protonation as well as methylation, delivering the desired product 233. The benzyl anion radical 238 could also be obtained by alkyl radical addition to electron-deficient indoles (Scheme 25c) and then undergo the CO_2_ trapping and subsequent transformation, furnishing functionalized indoline-3-carboxylic acids 236 and lactams 235.^83^

**Scheme 26:**
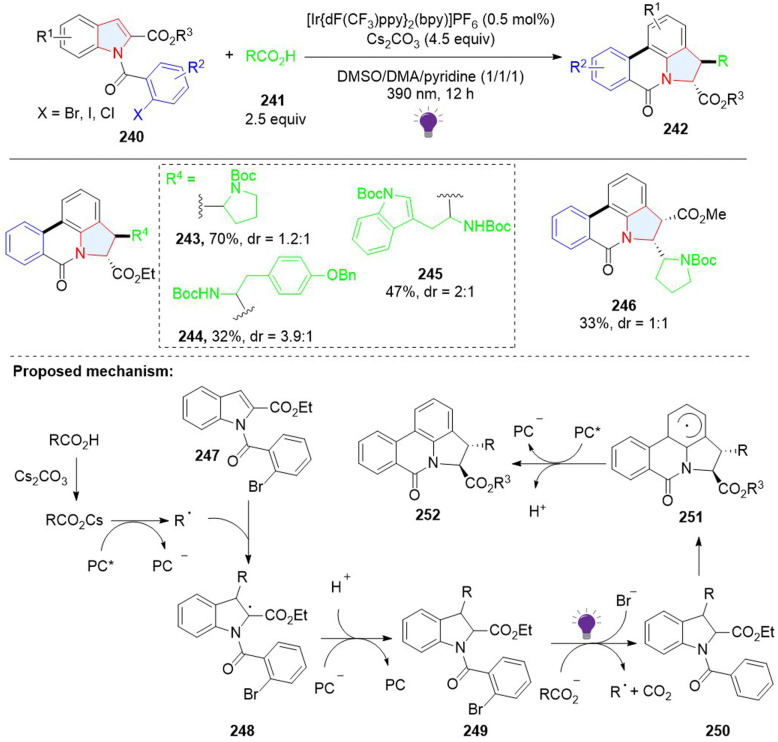
Photoredox catalysed stepwise, cascade dearomative 3,7-difunctionalization of indoles.

In addition to dearomative 2,3-difunctionalization of indoles, a 3,7-difunctionalization *via* a radical addition cascade was achieved by Sharma. A stepwise photoredox catalysed process gives polycyclic pyrrolophenanthridones 242 (Scheme 26).^84^ In the first step, the nucleophilic radical formed through decarboxylation of carboxylic acids was added to electron-deficient indole, giving 2,3-disubstituted indoline 249. Next, upon the irradiation of violet light, 249 was excited and underwent a bimolecular redox reaction with the carboxylate to afford the phenyl radical 250 with the release of Br^−^. The radical 250 addition to the C7 position of the indole ring delivered the arene radical 251, followed by sequential SET oxidation by the photocatalyst and deprotonation to obtain the final product 252.

**Scheme 27:**
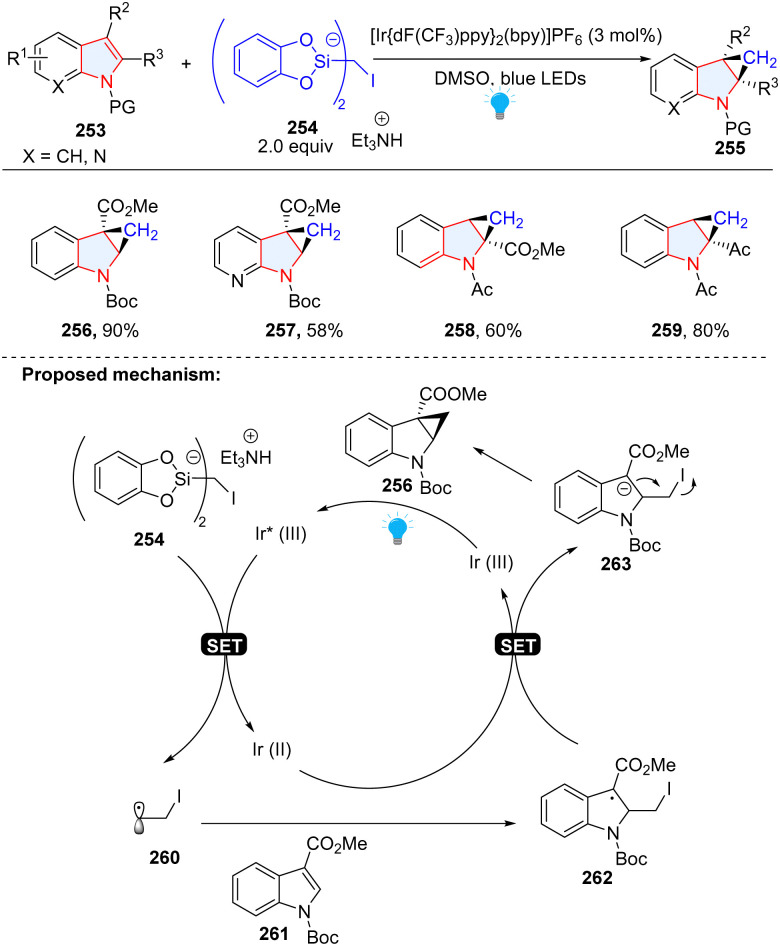
Photoredox catalytic cascade dearomative 2,3-difunctionalization of indoles for the synthesis of cyclopropane-fused indolines.

You, Cheng and co-workers disclosed the synthesis of cyclopropane-fused indolines *via* dearomatization of indoles with iodomethylsilicate 254 as a radical precursor (Scheme 27).^85^ Similar to our group's work, electron-deficient indoles 261 were employed as radical receptors. In this methodology, indoles were dearomatized by α-iodo alkyl radical 260 to give benzyl radical intermediate 262, followed by SET reduction to produce anion 263. The subsequent substitution delivered the final product 256.

**Scheme 28:**
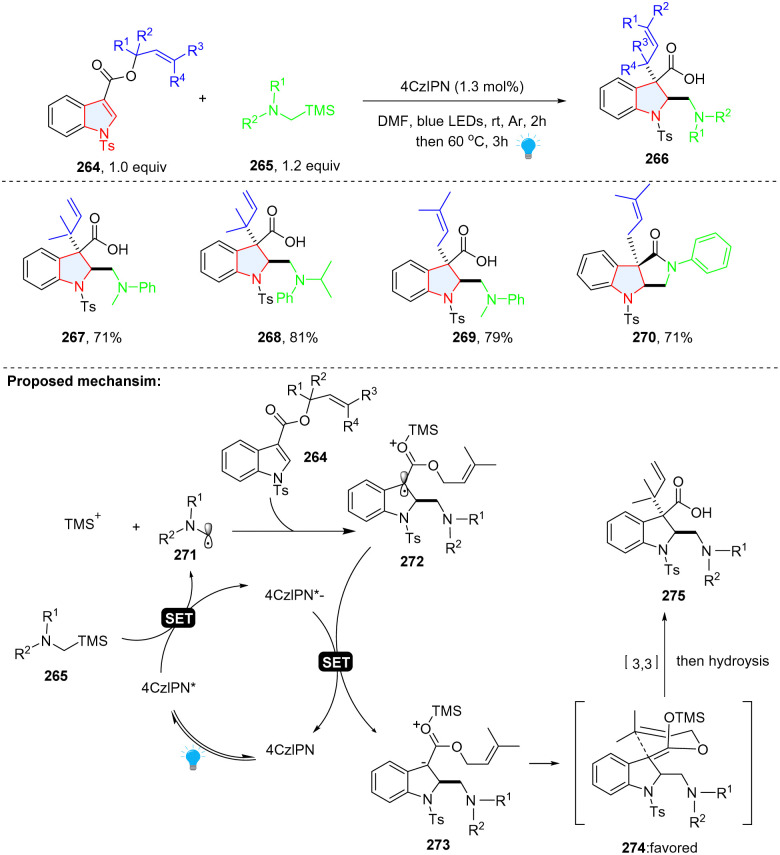
Photoredox catalysed cascade dearomative 2,3-difunctionalization of indoles *via* [3,3] rearrangement.

Prenylated and reverse-prenylated indolines are frequently present in numerous naturally occurring indole alkaloids.^86,87^ The traditional methods for the synthesis of such structures relied on transition-metal catalysis,^88–91^ which is limited to electron rich indole. Recently, Liu, Feng and co-workers documented the preparation of prenylated and reverse-prenylated indolines 266*via* photoredox catalysed Giese radical addition/Ireland–Claisen rearrangement from *N*-Ts prenyl/reverse-prenyl indole 3-carboxylate 264 (Scheme 28).^92^ Mechanistically, α-silylamine 265 was oxidized by excited state 4CzIPN, producing α-amino radical 271 and TMS^+^. Subsequently, radical addition to indole 264 and reduction of benzyl radical 272 led to the TMS^+^ activated carbanion 273, which underwent diastereoselective [3,3] rearrangement and generated prenylated indoline product 267–270 with a high dr value (>20 : 1).

#### 2.2.5 EnT promoted dearomative difunctionalization of heteroarenes *via* persistent iminyl radicals

**Scheme 29:**
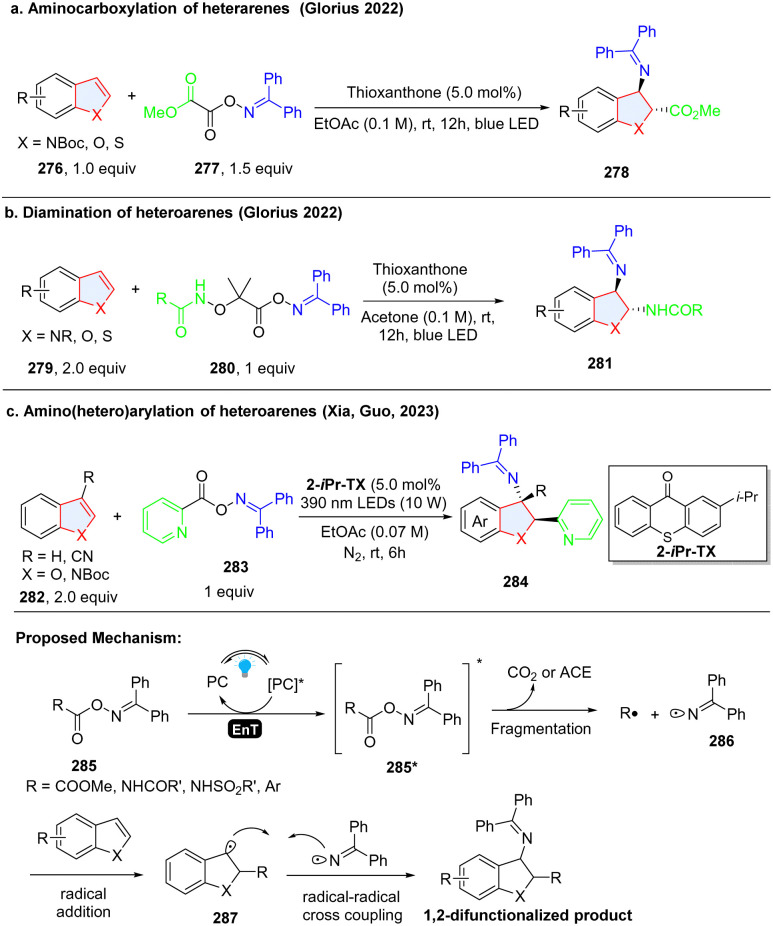
EnT facilitated cascade dearomative 2,3-difunctionalization of indoles/benzothiophenes/benzofuran by persistent iminyl radical trapping.

Although most of radical species are transient and highly unstable, the presence of an *in situ* formed persistent radical intermediate in a reaction system can trap the unstable and instantaneous radical species by facile radical–radical cross coupling.^93^ Oxime ester-based reagents were utilized for efficient and highly regioselective dearomative difunctionalization of indoles/benzothiophenes/benzofurans through the simultaneous generation of ambiphilic iminyl and electrophilic/nucleophilic radicals using an energy transfer strategy. In 2022, Glorius and coworkers designed and synthesized bench-stable bifunctional oxime oxalate esters as the precursors of both C-centred ester and N-centred iminyl radicals to prepare 2,3-disubstituted indoline *via* dearomatization of indole (Scheme 29a).^94^ In the proposed reaction pathway, methyl 2-(((diphenylmethylene)amino)oxy)-2-oxoacetate 285 (calculated triplet energy = 60.79 kcal mol^−1^) was excited to 285*via EnT by the excited thioxanthone (ET = 65.5 kcal mol^−1^). 285* underwent an N–O bond fragmentation followed by CO_2_ extrusion with a 5.5 kcal mol^−1^ barrier to generate the C-centred ester radical R˙ along with an N-centred iminyl radical 286. Then, R˙ was added to the C2 position of indole 276, producing benzyl radical 287. The stabilized radical intermediate 287 underwent a radical–radical cross-coupling process with the longer-lived N-centred iminyl radical 286 to deliver the desired 2,3-disubstituted indole compound 278. Shortly, the same group reported another oxime ester-based nitrogen-radical precursor 280, which enabled the unsymmetrical diamination of indoles/benzofurans/benzothiophens *via* EnT catalysed homolysis (Scheme 29b).^95^ Using the similar oxime reagent 283, Xia, Guo and coworkers prepared the 2-(hetero)aryl-3-amino-indoline 284*via* dearomatization of indole 282 (Scheme 29c).^96^

## 3 Photochemical dearomative skeletal editing of heteroarenes

The dearomative functionalization platform keeps the dearomatized aromatic skeletal (*e.g.*, size) unchanged, which narrows the complexity and diversity of newly established scaffolds. Recently, the dearomative skeletal editing, enabling the skeleton changes, is gaining significant interest. This type of transformation is particularly attractive in medicinal chemistry because it enables rapid transition of simple planar arenes into uncharted 3D chemical space, and the complex molecular architectures provide more potential for new structure discovery. In the past two years, photocatalyzed reactions have emerged as a powerful manifold for dearomative skeletal editing. Herein, we summarize these new findings. We classify these photochemical dearomative skeletal editing reactions into four categories, (1) dearomative cycloaddition, (2) dearomative ring expansion, (3) dearomative ring extraction, and (4) dearomative ring cleavage.

### 3.1 Photochemical dearomative photocycloaddition

The EnT-catalysed photocycloaddition of indoles process have been extensively studied by You,^97–100^ Fu,^101^ Dhar,^102^ Zhang,^103^ and Glorius.^104^ Several excellent reviews have covered the topics.^10,20,28,100,105^ The activated indoles exhibit triplet energy of 55–60 kcal mol^−1^, thus easily absorbing energy from visible-light-excited photocatalysts such as Ir(dF(CF_3_)ppy)_2_(dtbpy)(PF_6_), 60.8 kcal mol^−1^. They are readily promoted to the excited state through EnT, subsequently undergoing highly efficient dearomative cycloaddition. However, the dearomative cycloaddition of other heteroaromatics with higher triplet energy (Scheme 3), such as pyridine (79.4 kcal mol^−1^), furan (77.5 kcal mol^−1^), quinoline (62.4 kcal mol^−1^), benzofuran (71.9 kcal mol^−1^), *etc.* remain highly underdeveloped due to the shortage of efficient activation mode. With the advancement of photocatalysis, many new reactivities or strategies have been uncovered and applied to the dearomative cycloaddition of such heteroaromatics. In this section, we mainly focus on these newly reported photo-cycloaddition reactions for the less reactive heterocycles except the well-studied indoles.

#### 3.1.1 Intramolecular dearomative cycloaddition of activated alkene tethered heteroarenes

**Scheme 30:**
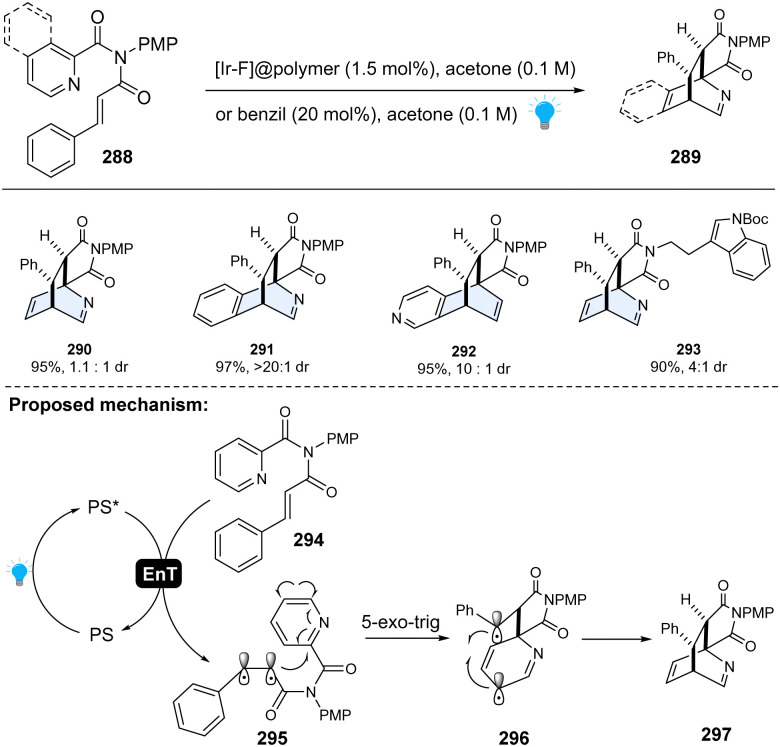
EnT activated-intramolecular dearomative cycloaddition of inert pyridines/isoquinolines.

To achieve selective dearomative photocycloaddition of heteroarenes, one feasible strategy is to tether such heterocycles to the activated alkenes (48–62 kcal mol^−1^), which can be activated through visible-light-mediated EnT to generate reactive biradicals for 1,2-biradical dearomative cycloaddition with arenes. Importantly, the dearomatized products are expected to be stable under the mild EnT-catalysed reaction condition. Glorius and colleagues successfully realized this concept (Scheme 30). Specifically, using the heterogeneous [Ir–F]@polymer as an EnT photocatalyst, the pyridine-containing cinnamyl amides 288 could be efficiently converted to the dearomative [4+2] cycloaddition products 289.^106^ No [2+2] or [3+2] cycloaddition product was observed, indicating the high regioselectivity. Mechanistically, once completing energy transfer from the excited state of the photocatalyst, the starting material 294 is promoted to triplet state – diradical intermediate 295. Subsequently, the electrophilic α-carbonyl radical undergoes 5-exo-trig cyclization to the pyridine moiety, giving rise to a 1,6-biradical species 296. Subsequent thermoneutral intersystem crossing into the open-shell intermediate 296 initiated the radical–radical recombination, affording the [4+2] cycloaddition product 297. Moreover, the polymer immobilized photocatalyst could be recycled, demonstrating the sustainability of this method. This pioneering work is inspirational because it suggests the inert heteroarenes can be dearomatized by rational design with energy-transfer activation.

**Scheme 31:**
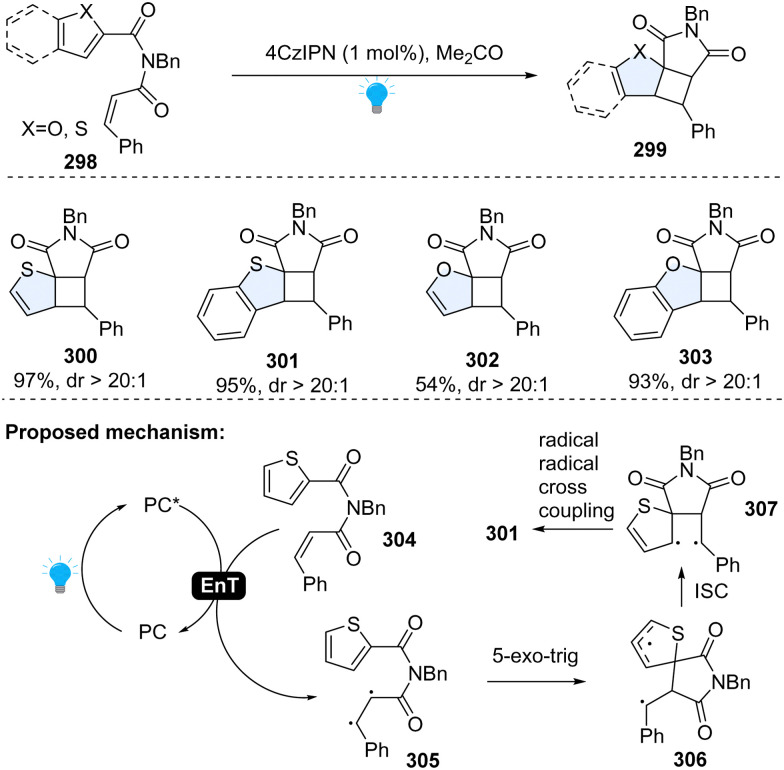
EnT catalysed-intramolecular dearomative cycloaddition of inert (benzo)thiophenes/(benzo)furans.

Later, a similar dearomative cycloaddition of inert heteroarenes including benzothiophene, benzofurans, thiophenes, and furans was achieved by Yin, Cao and Wang (Scheme 31).^107^ It is worth noting that even the highly inert thiophene or benzene works smoothly with this protocol. Specifically, these inert arenes were connected to styrene *via* an imide linker. Upon the irradiation of blue LEDs, photosensitizer 4CzIPN was excited to its triplet state. The energy transferred from irradiated 4CzIPN to the cinnamic moiety 304, forming the 1,2-diradical intermediate 305. The first C–C bond formation *via* 5-exo-trig radical cyclization, resulting in spiro intermediate 306. The spiro intermediate 306 was converted into the open-shelled singlet state 307, and finally furnished the desired product 301 through the radical combination.

#### 3.1.2 Intermolecular photocycloaddition of heteroaromatics

**Scheme 32:**
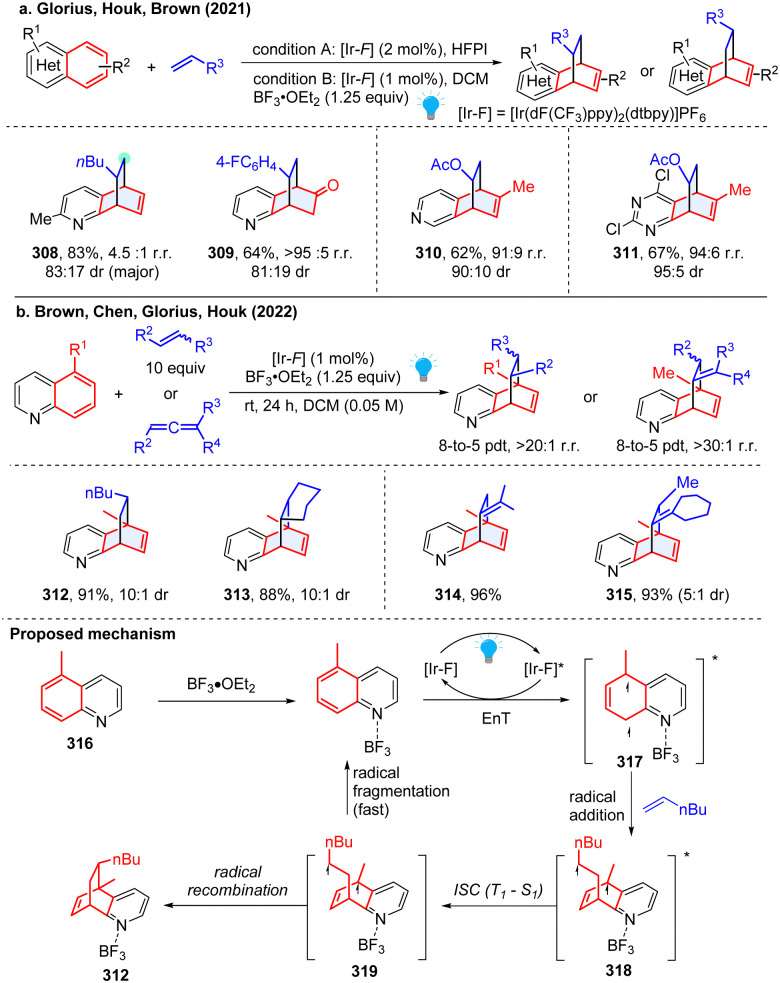
EnT promoted-intermolecular dearomative cycloaddition of inert bicyclic azoarenes.

Although the aforementioned intramolecular dearomative cycloaddition features high efficiency and reactivity, the intermolecular reactions are endowed with many advantages especially facial synthesis of substates and comparably high generality. However, the intermolecular reactions are underexplored because of the difficulty in controlling the regio- and diastereo-selectivities. Particularly, compared to the easily photosensitized indole and naphthalene, the abundant bicyclic azoarenes ((iso)quinoline, quinoxaline, quinazoline, *etc.*) require high activation triplet energy (>62.4 kcal mol^−1^) and possess higher resonance stabilization energy (quinone, 81 kcal mol^−1^; quinazoline 76.5 kcal mol^−1^), which is beyond the triplet energy of a common photosensitizer, and thus shows very limited application in the cycloaddition reaction. In 2021, Glorius reported an unprecedented energy transfer catalysed [4+2] dearomative cycloaddition of bicyclic heterocycles with a plethora of unactivated alkenes (Scheme 32a), providing valued bridged polycycles that previously have been inaccessible or required tedious synthetic efforts.^108^ Moreover, this reaction is highly regio- and diastereoselective, and can be applied to diverse transformations. Both experimental and computational studies validated the pivotal role of Brønsted and Lewis acids on the reaction efficiency and selectivity. The density functional theory calculations reveal that the triplet energy of quinoline decreases from 61.7 kcal mol^−1^ to 57.5 kcal mol^−1^ after protonation by HCl, rendering the substrates more amenable to EnT by photosensitizer (60.8 kcal mol^−1^). Regioselectivity is dependent on the transition state and solvent stabilization. Similarly, the Morofuji and Kano group independently reported a similar method, but proposed a different protonation role on the cycloaddition.^109^ Later, the Guin group presented a simplified protocol for the dearomatization of bicyclic azoarenes with alkenes using trifluoroacetic acid (TFA) as the Brønsted acid. This reaction is applicable to a broad range of substrates and works smoothly under aerobic conditions.^110^

It is noted that the above photochemical dearomative cycloadditions of bicyclic azoarenes with alkenes were mostly limited by the narrow substrates (non-5-substituded quinolines and terminal alkenes) and relatively poor regioselectivity. In 2022, the work by Houk and coworkers achieved highly regio- and stereoselective dearomatization cycloaddition with 5-substituded quinoline and highly substituted alkenes and allenes (Scheme 32b), generating sterically congested products 312–315.^111^ Based on the experimental and DFT studies, a reversible radical addition and a selectivity-determining radical recombination was revealed (Scheme 32). Similar to Morofuji and Kano groups’ proposal,^109^ the authors confirmed that the Lewis acid coordination is primarily associated with the radical addition step based on the investigated experiments.

**Scheme 33:**
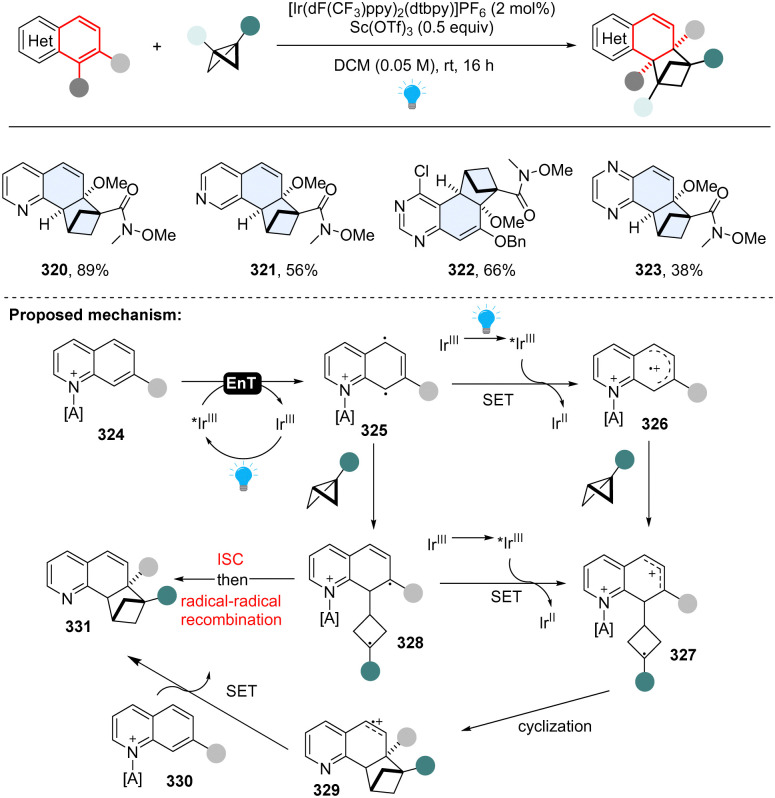
EnT catalysed-intermolecular dearomative [2π+2σ] cycloaddition of inert bicyclic azoarenes with bicyclo[2.1.1]butanes.

All these aforementioned methods prefer to form *para*-cycloaddition products since the *ortho*-selective reaction often triggers undesired consecutive rearrangement. Intriguingly, in 2023, Glorius and Houk disclosed an *ortho*-selective intermolecular dearomative [2π+2σ] photocycloaddition of bicyclic azoarenes with bicyclo[2.1.1]butanes (BCB) *via* a strain-release strategy (Scheme 33).^112^ In the study, the unexpected reactivity enables straightforward access to conformationally restricted bicyclo[2.1.1]hexane (BCH) frameworks (320–323). Computational studies suggest a distinct chain propagation mechanism. Upon EnT from photo-excited Ir(dF(CF_3_)ppy)_2_(dtbpy)*, the Lewis acid coordinated quinoline 324 is promoted to the excited state 325, which is easily oxidized by the excited Ir(dF(CF_3_)ppy)_2_(dtbpy)* and generates radical cation 326. Because the highest spin density is located at the C8-position, the carbon at C8 position selectively adds to BCB at the C3 position, yielding the cyclobutene radical cation 327. Alternatively, the cyclobutene radical cation could also be generated by oxidization of diradical intermediate 328*via* radical addition to BCB. The radical cation 327 undergoes cyclization and subsequently forms BCH radical cation 329. Finally, oxidation of neutral quinoline propagates the radical chain by releasing product 331 and another radical cation intermediate 326.

#### 3.1.3 Photoredox catalysed dearomative cycloaddition of heteroarenes

Energy transfer catalysis has been extensively explored in the dearomative photocycloaddition of indoles and inert azoarenes (pyridine, quinoline, thiophene, *etc.*). However, the photoredox catalysed dearomative cycloaddition of heterocycles (especially for inert bicyclic azoarenes) remains elusive due to the relatively complex tandem cycloaddition mechanism, possible incompatibility to redox reactivity, and issues of selectivity. In the past five years, various synthetic methods have been reported for the dearomative photocycloaddition of indoles, providing new insights and strategies which might be appliable in inert azoarenes. In this section, we will describe these newly reported methods and hope it will inspire more interest in developing new dearomatization approaches for the inert azoarenes.

**Scheme 34:**
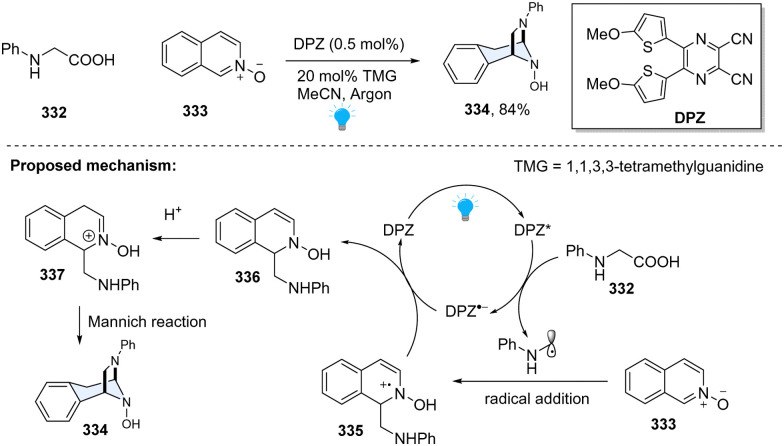
Photoredox catalyzed dearomative [3+2] cycloaddition of isoquinoline *N*-oxide.

In 2019, Jiang and coworkers disclosed a photoredox-catalysed formal [3+2] cycloaddition of *N*-aryl α-amino acids 332 with isoquinoline *N*-oxides 333 (Scheme 34).^113^ Inspired by the Minisci-type reaction of α-amino acid-derived redox-active esters with isoquinolines, it was envisioned that the key N-centred radical cation from Minisci-reaction could be reduced by a SET process, resulting in a dearomatized product. However, this proposal is very challenging since the aromatization of radical addition intermediate is thermodynamically favourable. In fact, the replacement of isoquinoline with *N*-oxide compound makes radical addition induced radical cation 335 easier to reduce. Meanwhile, the generated enamine 336 tends to undergo protonation, furnishing the iminium 337, which readily cyclizes with the amino group *via* the Mannich reaction. Cyclized product 334 is fully saturated and thus thermodynamically stable. This method enables facile access to diazabicyclo[3.2.1]octane-based *N*-heterocyclic compounds, providing a new approach to the dearomatization of challenging isoquinolines.

**Scheme 35:**
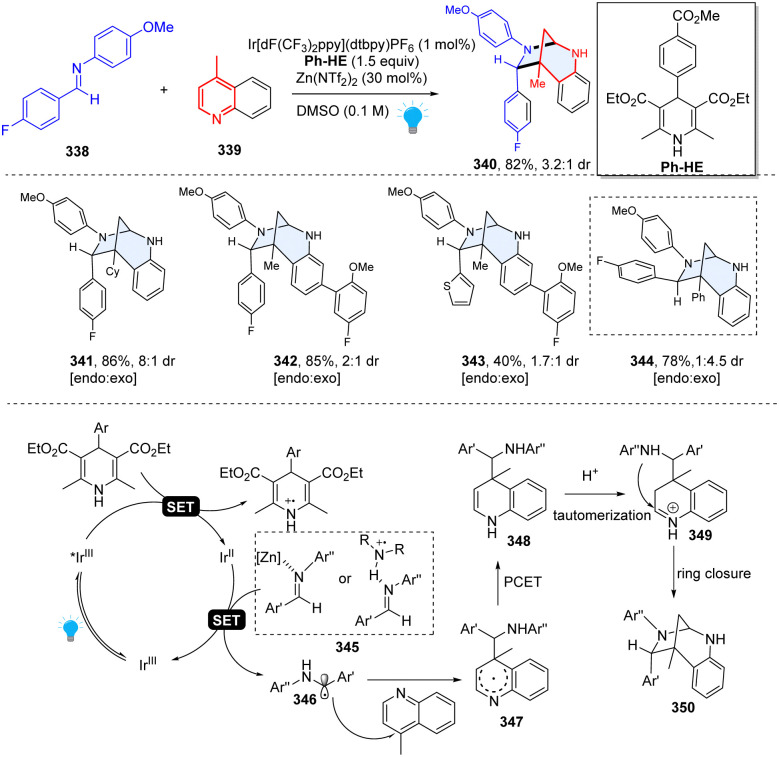
Photoredox catalyzed dearomative [5+2] cycloaddition of quinoline for the synthesis of 1,3-diazepanes.

In 2020, Dixon and Duarte described an unprecedented photoredox catalysed dearomative [5+2] cycloaddition for efficient construction of bridged 1,3-diazepanes (Scheme 35).^114^ Interestingly, this research was discovered serendipitously when originally studying the Minisci-type reactions between imine and quinoline. This method had excellent functional group tolerance and was regioselective for C–C bond formation at C4 because of the lower barrier to fragmentation than to any productive formation of C2-regioisomeric products. Mechanistically, the net reducing condition is prone to form C4-addition quinoline radical intermediate 347, which undergoes the proton-coupled electron transfer (PCET) and delivers the dearomatized dihydropyridine species 348. The dihydropyridine 348 is readily protonated to give iminium ion 349 under acidic conditions, subsequently followed by intramolecular cycloaddition to close the ring. This unusual reactivity provided a valuable option in the rapid synthesis of complex sp^3^-rich heterocycles.

#### 3.1.4 Direct UV light induced dearomative cycloaddition of five-membered heteroarenes

**Scheme 36:**
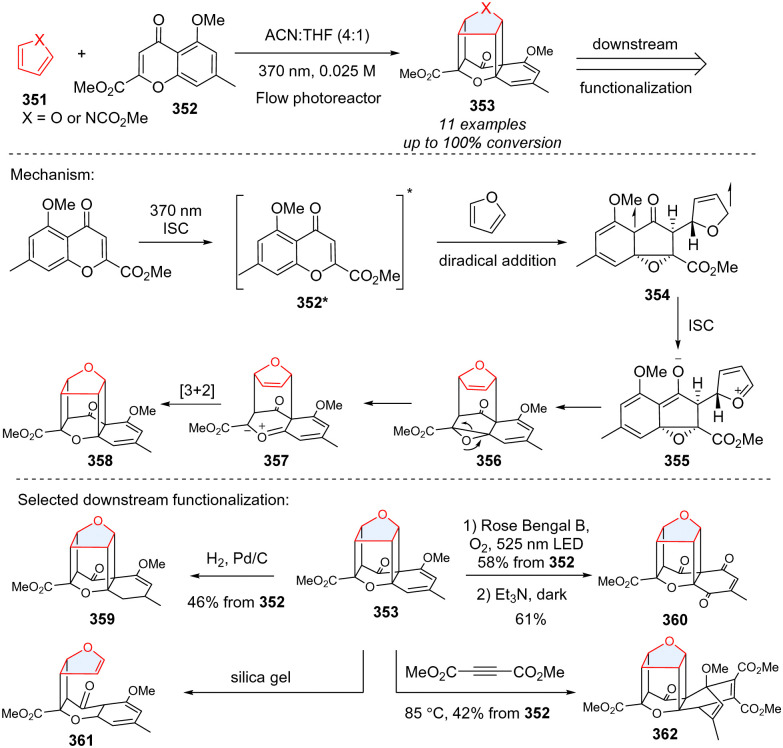
Photochemical intermolecular triple-dearomative cycloaddition of furan and pyrrole for the synthesis of caged scaffolds.

Intermolecular photocycloaddition of single five membered-heteroarenes (furan, pyrrole) is synthetically challenging considering their high triplet energy. Recently, Porco and coworkers serendipitously achieved triple-dearomative photocycloaddition of furans and pyrroles for creating an intriguing caged molecular framework (Scheme 36),^115^ which has potential applications in drug discovery and medicinal chemistry. Regarding the mechanism, the UV light (370 nm) can excite chromone 352, generating triple excited state 352*. Subsequently, the excited chromone 352* reacts with furan to generate a triplet biradical species 354 after dearomative cyclization, followed by intersystem crossing (ISC) to form the zwitterionic intermediate 355. The intermediate 355 undergoes cyclization to which may subsequently ring-open to carbonyl ylide 357, followed by intramolecular [3+2] cycloaddition to caged cycloadduct 358. The caged product 353 can undergo diverse downstream functionalization including hydrogenation (359), photoredox catalysed oxidation (360), retrocyclization (361), and Diels–Alder reaction (362).

### 3.2 Photochemical dearomative ring expansion of heteroaromatics

Seven-membered heterocycles, such as azepines,^31,116^ oxepines,^117^*etc.* are broadly featured in pharmaceuticals. However, the synthesis of such molecular scaffolds mainly rely on the [4+3]^118,119^ and [5+2]^120,121^ cycloadditions or elaborately designed *de novo* syntheses.^122–124^ These methods have limitations in scope and utility, and require multistep synthesis of precursors. Newly emerged photochemical dearomative ring expansion provides a simple and efficient way to rapidly construct these ‘privileged’ seven membered rings from inert heteroarenes.

#### 3.2.1 Direct photoactivated dearomative ring expansion of heteroaromatics

**Scheme 37:**
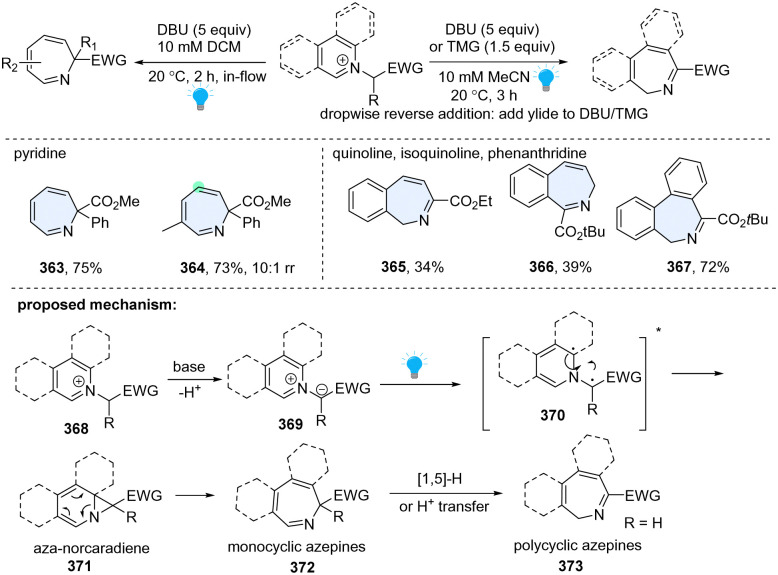
Direct photoactivated dearomative ring expansion of heterocycles by insertion of one C atom.

In 2021, the Beeler group developed a unified approach to the synthesis of seven-membered azepines by photosensitized dearomative ring expansion of diverse azoarenes (Scheme 37).^125^ Inspired by the photolysis of *N*-iminopyridinium ylides by UV light, they directly employed pyridinium salt 368 and strong base 1,8-diazabicyclo[5.4.0]undec-7-ene (DBU), which facilitated *in situ* formation of the azomethine ylide 369. The resulting ylide 369 could efficiently undergo blue light photolysis in a flow reactor, delivering the desired ring expansion products 363–367. Other than monocyclic azepine products (363, 364), this protocol is also applicable to quinoline (365), isoquinoline (366), and phenanthridine (367), providing polycyclic azepines. As to the mechanism, upon excitation by blue LEDs, the heteroaromatic *N*-ylide 369 is promoted to its singlet state, diradical intermediate 370, followed by radical recombination and 6π-electrocyclic ring opening, giving azepine 372. When the R group is hydrogen for the polycyclic arenes, it is prone to undergo the subsequent [1,5]-H transfer or proton transfer, generating the α-imino ester isomer 373.

**Scheme 38:**
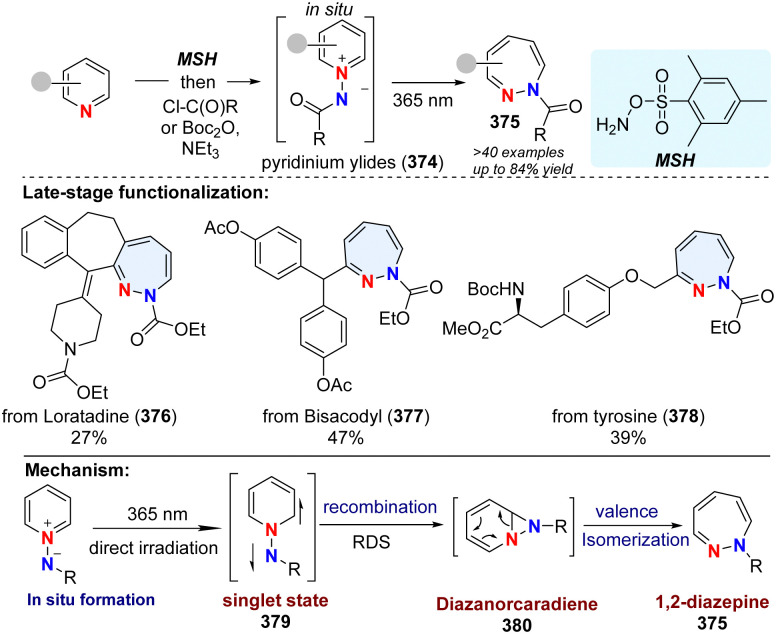
Photochemical dearomative skeletal enlargement of pyridine for the synthesis of 1,2-diazepines by insertion of one N atom.

In addition to the insertion of one C atom into pyridine, 1-aminopyridinium ylide 374 was reported to undergo photochemical rearrangement, giving 1,2-diazepines 375 by insertion of one N atom (Scheme 38).^126^ However, the works are limited to simple substrates. Recently, Moreau and Ghiazza coworkers developed a one-pot procedure, converting diverse pyridine derivatives into 7-memberrd 1,2-diazepines including some pharmaceuticals derived analogues (376–378).^127^ Notably, the diazanorcaradiene intermediate (380) is proved to be involved in the photochemical transformations. Mechanistically, the *in situ* formed pyridinium ylides 374 are directly activated by UV light, delivering singlet state intermediate 379. After recombination, the rate determining step, the diazanorcaradiene intermediate 380 rapidly undergoes ring opening to afford the desired product 375.

#### 3.2.2 Photoactive arenophiles induced dearomative ring expansion

**Scheme 39:**
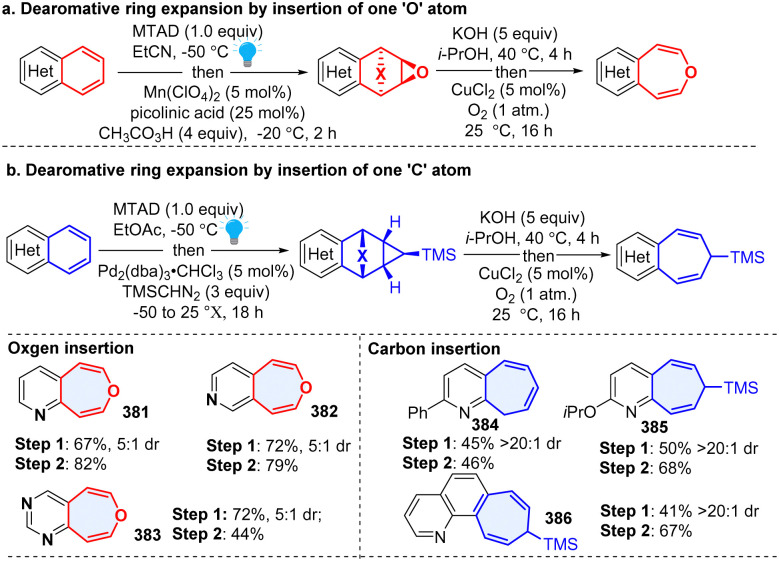
Photoactivated arenophiles enabled dearomative ring expansion of heteroarenes.

An arenophile-based dearomative strategy has great potential for diverse downstream transformations once the visible-light triggered cycloadduct is generated. In 2020, Sarlah and coworker developed a versatile protocol to synthesize oxepines through insertion of an ‘O’ atom to inert bicyclic azoarenes (Scheme 39), including quinoline, isoquinoline, and quinazoline, which delivered azobenzoxepines (381–383).^128^ Two years later, the same group reported a general arenophile-based method for the synthesis of pharmaceutically valued (aza)benzocycloheptenes (384–386) from polycyclic (hetero)arenes by dearomative insertion of a ‘C’ atom into the heteroaromatic.^129^ This synthetic approach is expected to have broad applications in drug discovery and development.

#### 3.2.3 Photoredox catalysed dearomative furan expansion *via* Achmatowicz rearrangement

**Scheme 40:**
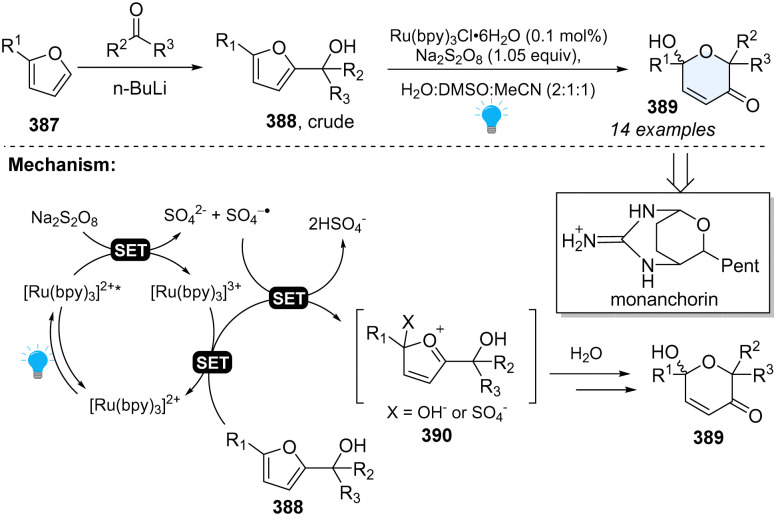
Photoredox catalysed dearomative ring expansion of furans.

Furfuryl alcohols are precursors to highly ornamented dihydropyranones *via* Achmatowicz rearrangement, which was first reported by Cavill and extensively studied by Achmatowicz.^130^ Until 2017, Gilmore reported the first photoredox-catalysed Achmatowicz reaction for the construction of functionalized dihydropyranones (Scheme 40).^131^ This protocol is quite robust and potentially appliable to the synthesis of natural product—Monanchorin. Regarding the mechanism, upon irradiation by blue LEDs, the excited photocatalyst will be quenched by Na_2_S_2_O_8_ first, generating Ru^III^, which cooperatively oxidizes the furan derivatives 388 with SO_4_^−^˙. Furans 388 will lose two electrons, furnishing oxocarbenium intermediates 390, followed by water addition and the loss of X to deliver the desired products 389.

#### 3.2.4 Photoredox catalysed dearomative ring expansion of (benzo)thiophenes by bicyclobutane insertion

**Scheme 41:**
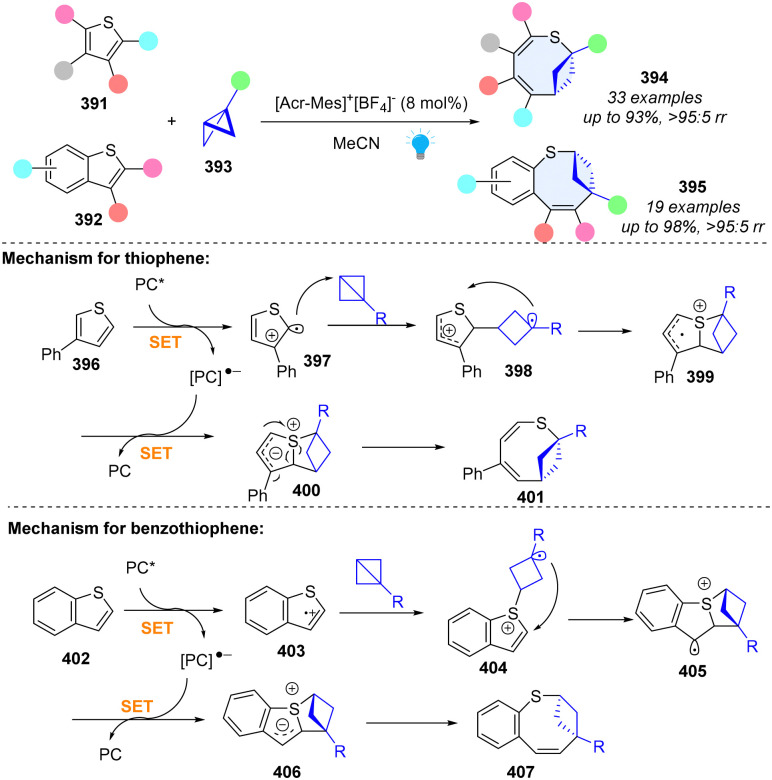
Photoredox catalysed dearomative ring expansion of (benzo)thiophenes by bicyclo[1.1.0]butane insertion.

Insertion of C(sp^3^)-rich rings into heterocycles to form bicyclic frameworks could enhance the sp^3^ richness, three dimensionality, and increased conformational rigidity, which is often useful in drug discovery.^1^ In 2023, Glorius and coworkers reported an unprecedented skeletal ring enlargement by bicyclo[1.1.0]butane insertion, producing an eight-membered bicyclic ring (Scheme 41).^132^ Importantly, this method is compatible with broad functional groups with excellent chemo- and regioselectivity, and the synthetic derivatization of this method demonstrates its synthetic value. For thiophenes, the excited PC* oxidizes the thiophene to form the radical cation intermediate 397. Due to the highest spin density on C2, the BCB insertion occurs at the C2 position of thiophene and generates radical cation intermediate 398. Subsequent C–S bond formation generates heterocycle intermediate 399. Upon reduction by the photocatalyst (PC), the C–S bond cleavage gives the desired product 401. For benzothiophenes, the mechanism is slightly different due to the different spin density distribution of radical cation intermediate 403. Moreover, the BCB insertion into the S-radical of benzothiopene has a similar free-energy barrier (12.9 kcal mol^−1^) to the BCB insertion into the C-radical of thiophene (12.6 kcal mol^−1^). Subsequently, the C–C bond formation generates fused heterocycle radical cation 405, which undergoes the kinetically favourable C–S cleavage to obtain the desired product 407. We believe that the bicycle (‘ring-in-a-heterocycle’) design strategy will inspire more studies to construct pharmaceutically diverse heterocyclic compounds.

### 3.3 Photochemical dearomative ring contraction of heterocyclics

In contrast to the dearomative skeletal enlargement, dearomative ring contraction offers a distinct way for skeletal editing. It involves the exclusion of one or more atoms from the parent aromatics, and the newly established ring becomes C(sp^3^)-rich cycles. The saturated, rigid, and small rings are readily accessible from the simple (hetero)arenes. It is a highly underexplored field. To our knowledge, only two studies for heteroarenes have been reported so far.

#### 3.3.1 Dearomative ring contraction *via* pyridium photoaddition

**Scheme 42:**
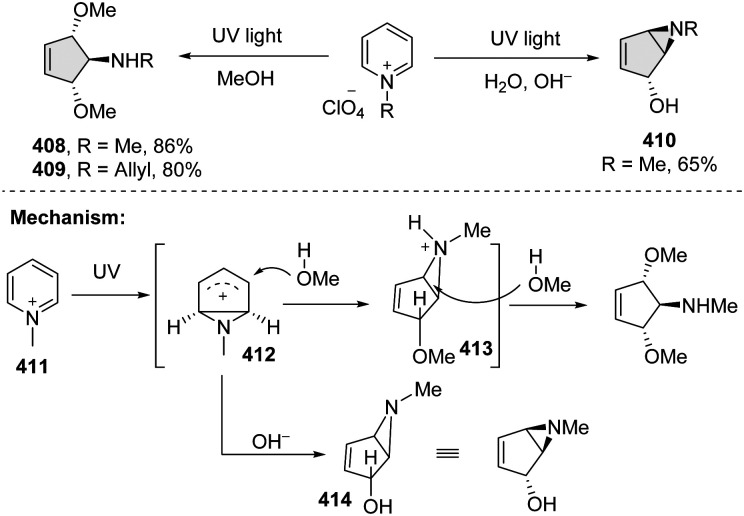
Direct UV light activated dearomative ring contraction of pyridiniums.

About 40 years ago, Mariano and coworkers pioneered a pyridinium photoaddition approach, which can access the highly functionalized cycloheptanes with excellent stereoselectivity from simple pyridines (Scheme 42).^133–135^ Importantly, this synthetic technique found its wide utilization in the total synthesis of natural products, such as mannostain A, trehazolamine, allosamizoline, and penoses, *etc.* Mechanistically, upon irradiation by UV light, the pyridinium salt 411 undergoes a photoelectrocyclization reaction, leading to the production of rigid cyclic cation intermediate 412. The cation intermediate proceeds with the nucleophilic addition in the presence of methanol, resulting in the protonated bicycloaziridine 413. Attack by the second methanol delivers the *trans–trans*-dimethoxycyclopentenyl amines 408–409. Under basic conditions, the cation intermediate 412 is attacked by hydroxide to give bicycloaziridine 414.

#### 3.3.2 Consecutive energy transfer induced cycloaddition/rearrangement reaction induced dearomative ring contraction

**Scheme 43:**
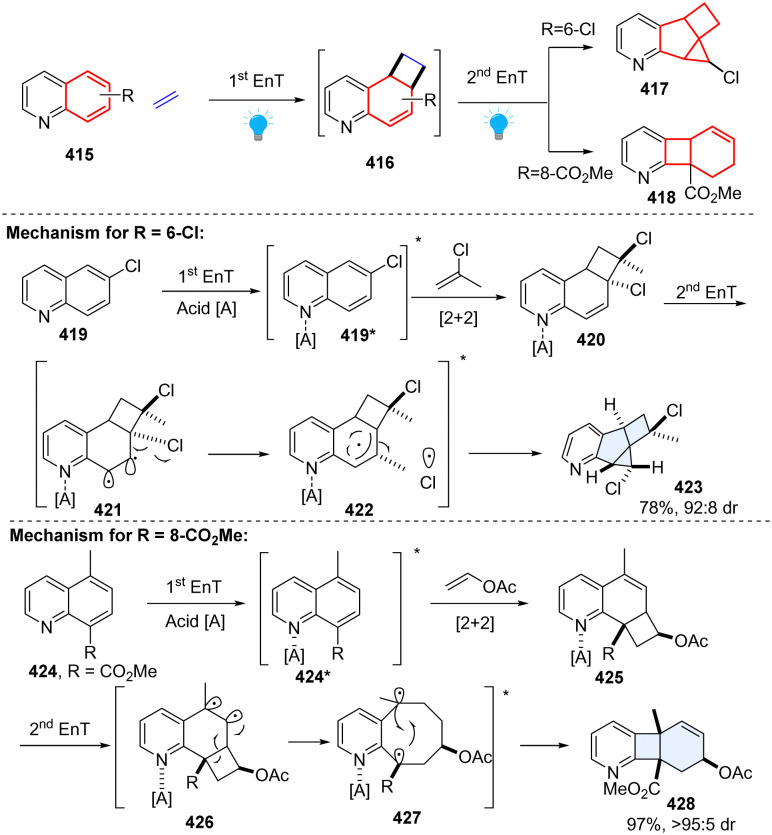
Consecutive EnT catalysed ring contraction of quinolines.

In 2022, Glorius elegantly presented two types of consecutive EnT-mediated dearomative ring contraction of quinoline by intermolecular sequential dearomative [2+2] cycloaddition/rearrangement reaction (Scheme 43).^136^ This protocol enables facile access to pharmaceutically relevant hybrid fused 2D/3D rings through one-pot operation. Specifically, under acidic conditions, 6-chloroquinoline 419 reacts with 2-chloropropene in the presence of the photosensitizer Ir[dF(CF_3_)ppy]_2_[dtbbpy](PF_6_), furnishing a structurally unique pyridine-fused 6-5-4-3 ring system 417 with high diastereoselectivity. While methyl quinoline-8-carboxylate 424 with vinyl acetate provides a new type of polycyclic product, a fused 6-4-6 ring system with one single diastereo-isomer 418. Regarding the mechanism, first EnT mediated dearomative [2+2] cycloaddition occurs under the irradiation of blue LEDs, generating cinnamyl chloride analogue intermediate 420. Subsequently, the second EnT event triggers a homolytic bond dissociation of C–Cl, furnishing a triplet radical pair 422, followed by C–C and C–Cl bond formations to deliver the terminal 6-5-4-3 ring product 423. As to the 6-4-6 ring product, the methyl quinoline-8-carboxylate 424 and vinyl acetate begin with a similar EnT-promoted *ortho*-dearomative [2+2] cycloaddition, resulting in a vinylcyclobutane intermediate 425. The consecutive EnT process initiates the subsequent cyclobutane rearrangement, forming a kinetically stable fused ring product 428.

### 3.4 Photochemcial dearomative ring cleavage

Dearomative ring cleavage usually involves the destruction of aromaticity and C–C or C–heteroatom bond breakage under harsh conditions. The traditional methods are limited to stoichiometric transition-metal complex or enzymes in bacteria.^137–139^ In 2021, Jiao and Houk reported a general, copper-catalysed arene-ring cleavage method for access to alkenyl nitriles, which shows the broad utility in the late-stage functionalization of inert (hetero)arenes.^140^ Recently, with the development of photocatalysis, some visible-light-mediated dearomative ring opening methods arise. However, these methods are only limited to specific heteroarenes and reaction types. Herein, we present the progress on this topic and expect more general strategies to emerge in the future.

#### 3.4.1 Photoredox catalysed ring cleavage of thiophene/furan

**Scheme 44:**
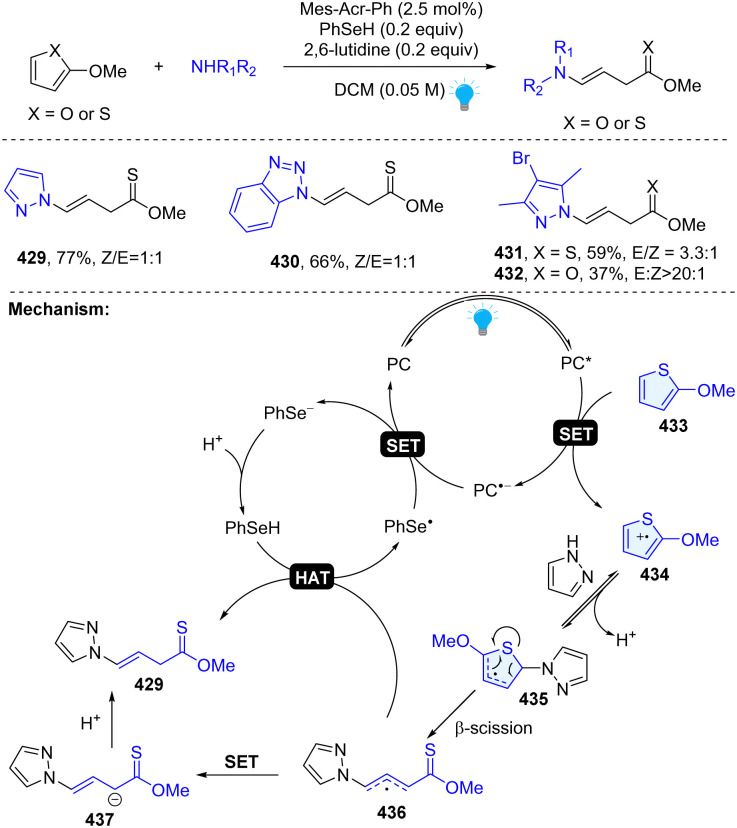
Photoredox catalysed dearomative ring cleavage of thiophenes/furans.

In 2023, our group uncovered a photoredox catalysed dearomative ring cleavage of electron rich thiophenes and furans for the construction of synthetically challenging thionoester or ester with a vinyl group (Scheme 44).^33^ This reaction is compatible with diverse pharmaceutically important azoles and appliable to the late-stage functionalization of drug-like molecules. Mechanistically, 2-methoxy-thiophene could be oxidized by excited state PC* (
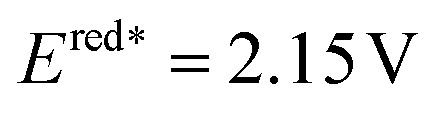
), resulting in the thiophene radical cation intermediate 434. Subsequently, the pyrazole attacks the C2 position and generates thiophene radical intermediate 435 after simultaneous deprotonation. Unexpectedly, the thiophene radical intermediate 435 undergoes a ring opening β-scission and furnishes the desired product 429 through either an HAT process or ET/PT process.

#### 3.4.2 Photoredox catalyzed dearomative C–N cleavage of indole

**Scheme 45:**
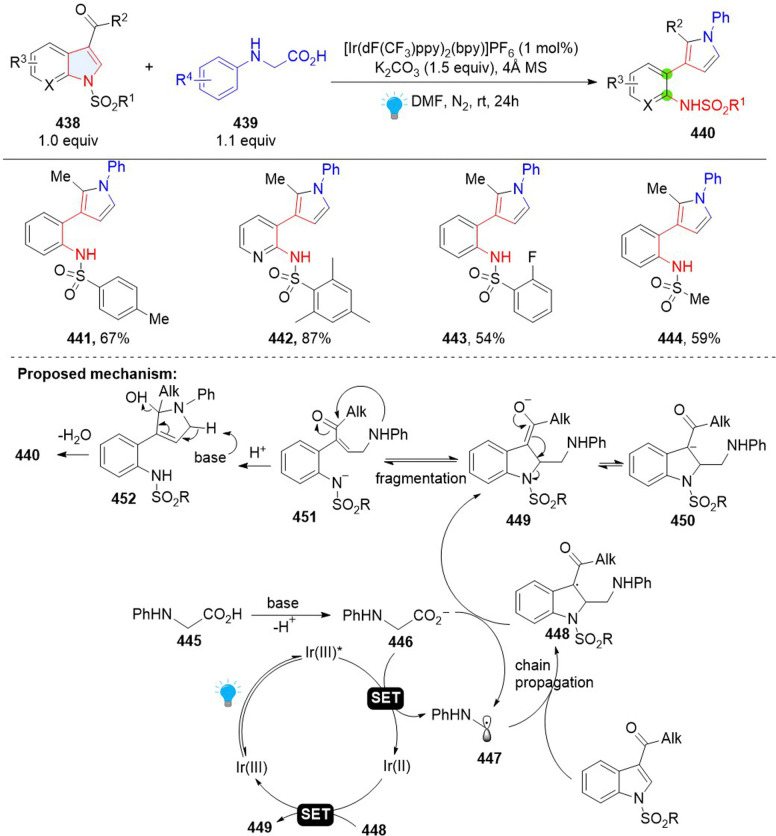
Photoredox catalysed dearomative ring cleavage of indoles.

Recently, Wang and coworkers developed the synthesis of 3-(*o*-aminophenyl)pyrroles by remodelling of *N*-sulfonyl-3-acyl indoles 438 with *N*-phenylglycines (Scheme 45).^141^ This is a Giese type/ring opening cascade reaction. Similar to the previous work, the radical addition between indole 438 and nucleophilic radical 437 gives benzyl radical intermediate 448, which was reduced by Ir(ii) or glycine anion producing benzyl anion 449. Then intramolecular retro-aza-Michael reaction delivers γ-amino unsaturated ketone 451 and nucleophilic addition followed by dehydrative aromatization, leading to the final product 440.

#### 3.4.3 Photochemical dearomative ring opening of pyridine

**Scheme 46:**
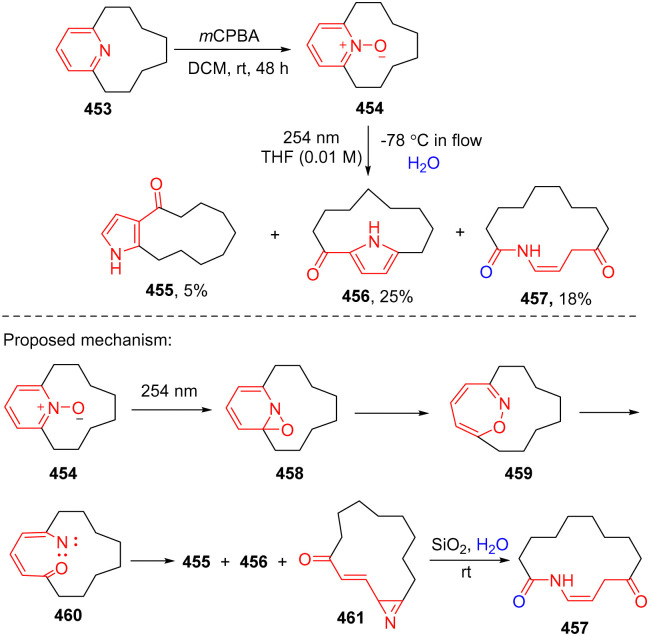
Photochemical dearomative ring opening of pyridine *N*-oxide.

The direct photoactivated ring of pyridines is challenging considering its high triplet energy (79.4 kcal mol^−1^). An alternative strategy for photo-activation of the pyridine ring is to sensitize its oxidized counterpart – pyridine-*N*-oxide to undergo ring contraction.^142^ Recently, Harran and coworkers reported an improved pyridine-ring rearrangement of the *N*-oxide 454 leading to the formation of ring contracted ketopyrrolophanes 455 and 456 and a new ring opening macrolactam 457 (Scheme 46).^143,144^ The DFT calculation was performed to rationalize the observation of the formed lactam.^144^ The UV light excited *N*-oxide thermodynamically favours the C–O bond formation to give oxaziridine 458. Cleavage of the C–N bond occurs with a low barrier of 4.5 kcal mol^−1^, generating 1,2-oxazepine 459. Breaking the N–O bond by photochemical activation of the oxazepane delivers singlet state nitrene 460, which is transformed into ketopyrrolophanes 455 and 456 and a new azirene 461. The resulting azirene is hydrolysed to give the ring opening product 457.

## 4 Conclusions

For only over five years, we have seen significant developments in photochemical dearomatization. A number of innovative and useful methods have been developed and provided an efficient synthetic platform to construct the 3D molecular architectures. The strategies of photochemical dearomative functionalization and skeletal editing enable rapid assembly of molecular complexity and diversity. The impressive developments are attributed to expanding photochemical activation modes, harnessing new radical reactivities, understanding selectivity and *de novo* designing substrates. Diverse unprecedented transformations of the photochemical dearomative functionalization have been realized. They include photoredox catalysed hydrofunctionalization, photoredox-induced radical addition to electron deficient arenes, arene radical and alkyl radical cross-coupling, direct photoexcitation of arene or arenophiles induced dearomatization, *etc.* The dearomative skeletal editing has been accomplished *via* EnT mediated photocycloaddition, photoredox catalysed rearrangement or cascade reactions, direct photoexcitation induced arenes substrates homolysis or arenophiles induced skeletal modifications, *etc.*

**Scheme 47:**
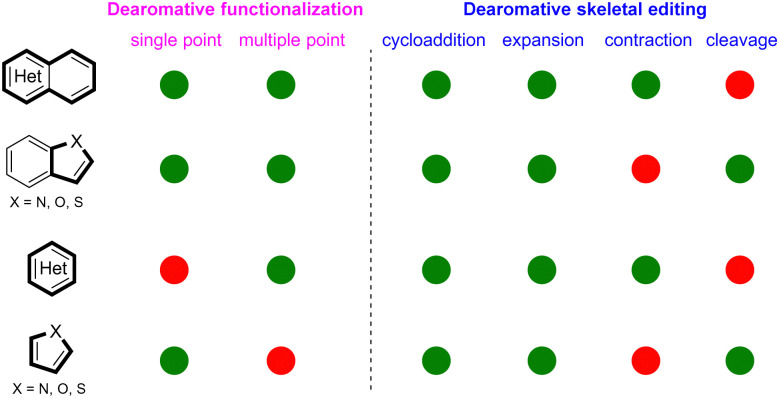
Qualitative description of the development of dearomative skeletal modifications for different types of heteroarenes. The green-coloured circles mean the existence of initial well-developed research. The red-coloured circles represent the non-existence of well-developed research.

Despite the tremendous progress of photochemical dearomatization, many unmet synthetic challenges remain. Dearomative functionalization is largely applied to bicyclic azaarenes ((iso)quinoline, quinaxaline, quinozoline, *etc.*) or five-membrane fused heteroaromatics (indole, benzothiophene, benzofuran, *etc.*). However, the application of the strategy for single ring heteroarenes such as pyridine, thiophene, furans, pyrrole, *etc* is still limited (Scheme 47, red circles). Regarding dearomative skeletal editing, although many common heteroaromatics can achieve dearomative photocycloaddition and photochemical ring expansion (Scheme 47, green circles), the photochemical dearomative ring contractions are limited to polycyclic azoarenes or six-membered single ring heteroarenes, and the photochemical dearomative ring cleavages are confined to the very few five-membered single rings (thiophene/furan) or indoles. Additionally, the photoinduced asymmetric dearomative modifications are a largely untapped field. As an efficient way to construct enantioselective sp^3^ rich heterocycles from heteroarenes, the cooperative photoredox- and bio-catalysed dearomatization may be desirable, but research in this field is limited by the generality and feasibility. Finally, considering the broad redox potential spectrum, electrophotochemistry^145,146^ is expected to harness its unique strength in the dearomatization of inert heteroarenes.

With the recent breakthroughs of skeletal editing for rapid construction of new complex frameworks,^147^ we foresee that the combination of photoredox catalysed dearomatization with molecular skeletal editing^148^ might provide an opportunity for developing new dearomative skeletal editing strategies. Moreover, leveraging the traditional dearomative ring cleavage and photoredox catalysis will inspire synthetic chemists to investigate more novel strategies. More general new photochemical methods, especially for dearomative ring contraction and cleavage, are expected to flourish and thus provide new methods for dearomative skeletal modifications, which will find broad application in the total synthesis of natural products and pharmaceutically related molecules.

## Conflicts of interest

There are no conflicts to declare.
